# Deep thermal profiling for detection of functional proteoform groups

**DOI:** 10.1038/s41589-023-01284-8

**Published:** 2023-03-20

**Authors:** Nils Kurzawa, Isabelle Rose Leo, Matthias Stahl, Elena Kunold, Isabelle Becher, Anastasia Audrey, Georgios Mermelekas, Wolfgang Huber, André Mateus, Mikhail M. Savitski, Rozbeh Jafari

**Affiliations:** 1 Genome Biology Unit, European Molecular Biology Laboratory (EMBL), Heidelberg, Germany; 2 Institute for Research in Biomedicine (IRB Barcelona), Barcelona Institute of Science and Technology, Barcelona, Spain; 3 Clinical Proteomics Mass Spectrometry, Department of Oncology-Pathology Karolinska Institutet, Science for Life Laboratory, Solna, Sweden

**Keywords:** Mass spectrometry, Protein-protein interaction networks, Cancer therapy, Post-translational modifications, Networks and systems biology

## Abstract

The complexity of the functional proteome extends considerably beyond the coding genome, resulting in millions of proteoforms. Investigation of proteoforms and their functional roles is important to understand cellular physiology and its deregulation in diseases but challenging to perform systematically. Here we applied thermal proteome profiling with deep peptide coverage to detect functional proteoform groups in acute lymphoblastic leukemia cell lines with different cytogenetic aberrations. We detected 15,846 proteoforms, capturing differently spliced, cleaved and post-translationally modified proteins expressed from 9,290 genes. We identified differential co-aggregation of proteoform pairs and established links to disease biology. Moreover, we systematically made use of measured biophysical proteoform states to find specific biomarkers of drug sensitivity. Our approach, thus, provides a powerful and unique tool for systematic detection and functional annotation of proteoform groups.

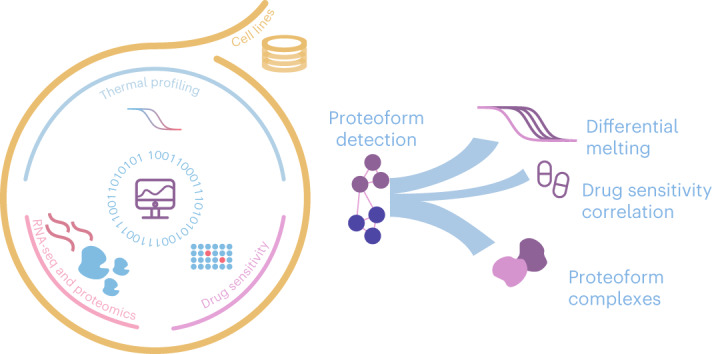

## Abstract (web summary)

Applying thermal proteome profiling to acute B cell childhood leukemia cell lines combined with deep peptide fractionation and a graph-based clustering algorithm allows inference of functional proteoform groups and their association with drug response.

## Main

Proteins are the functional units expressed from genes and ultimately define the phenotype of cells. Through genomic variation (that is, mutations and single-nucleotide polymorphisms), alternative splicing of transcripts, proteolytic cleavage, post-translational modifications (for example, phosphorylation, ubiquitination, acetylation and others) and protein–protein interactions (PPIs), the complexity of the functional proteome is expanded to millions of proteoforms^1,2^. Therefore, identification and functional characterization of proteoforms can improve understanding of biological processes in health and disease.

Although global proteoform measurement is critical for achieving full proteome characterization and annotation, its realization is still hampered by technological and analytical limitations. Top-down proteomics enables the precise characterization of proteoforms of individual proteins^3^, and inference based on peptide-level data from bottom-up proteomics has recently been established using different approaches^4,5^. In peptide correlation analysis (PeCorA)^4^, the pattern across samples of individual peptides mapping to a protein is compared to all other peptides to find differentially abundant peptides that may reflect proteoforms. Correlation-based functional proteoform assessment (COPF)^5^, however, aims to detect proteoforms supported by multiple peptides using a combination of PeCorA and hierarchical clustering, cutting obtained clusters to obtain a predefined number of proteoforms per protein that are tested for significance and scored by within versus across cluster correlation. However, these approaches, although powerful, can be limited by proteome coverage or by availability and variability of sample conditions that distinguish different proteoforms. Furthermore, proteoforms have been detected representing protein sequence and post-translational modification status differences, but other important variations of functional protein state, including protein complex and metabolite associations, are difficult to distinguish without specific targeted experimental methods and have, therefore, been excluded from identification. Recent initiatives have been proposed to define a human proteoform reference^2,6^, and a reference map of proteoforms of human hematopoietic cells has recently been reported^7^, and additional efforts are underway to address these gaps and improve knowledge of proteoforms.

Thermal proteome profiling (TPP) is a method originally developed for unbiased detection of drug targets in living cells^8^ and, more recently, tissues^9^ by monitoring the changes in the thermal stability of proteins upon drug binding. It is implemented by applying the cellular thermal shift assay (CETSA)^10^ on a proteome-wide scale using multiplexed quantitative mass spectrometry^11^. Recent work has shown that TPP can not only inform on drug–target engagement but also on protein–nucleic acid^12^, protein–protein^13^ and protein–metabolite interactions^14^ as well as metabolic pathway activity^15^ and the functional relevance of post-translational modifications^16^. Moreover, it has been found that cell-type-specific physiology is reflected in characteristic proteome thermal stability profiles and can be predictive of drug responses^17^.

Here we introduce the application of TPP for the detection of functional proteoform groups. We demonstrate this by applying TPP to detect biologically influenced melting differences without any drug perturbation in 20 different B cell childhood acute lymphoblastic leukemia (cALL) cell lines, representing various disease subtypes defined by characteristic chromosomal rearrangements. In combination with high-resolution isoelectric focusing fractionation (HiRIEF)^18^, we measured thermal stability with unprecedented peptide coverage per gene. This aspect was exploited to infer functionally relevant proteoform groups in an unbiased manner, capturing differently spliced, modified or cleaved proteins expressed from the same gene. We linked differentially thermally stable proteoform groups across cell lines with the developmental stage of the cell of origin and the genetic subtypes of the cALL samples. Moreover, we analyzed differential co-aggregation of pairs of proteoform groups across the different cALL cell lines and linked co-aggregation to disease biology. Lastly, we systematically made use of measured biophysical proteoform states to find biomarkers for cell line sensitivity to 528 oncology and investigational compounds. The results of protein and proteoform group melting can be explored and used for hypothesis generation in a user-friendly online tool at https://www.proteomics.se/deepmeltome/.

## Results

### Deep thermal profiling assigns peptides to proteoforms

**Fig. 1:**
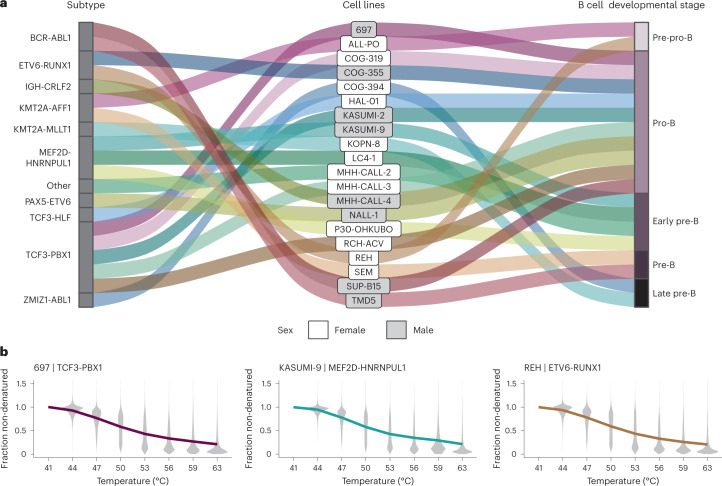
Characteristics and thermal profiles of cALL cell lines in this study. **a**, Alluvial diagram representing profiled cell lines and their characteristics. **b**, Exemplary average melting profiles across all peptides identified and quantified in the cell lines 697, KASUMI-9 and REH after normalization. Source data

To systematically measure the melting behavior of proteins in cALL cell lines representing different molecular subtypes, we performed temperature-range TPP^8^ with eight temperatures per sample and multiplexed two cell lines at a time using TMTpro^19,20^ (Supplementary Table 1). We profiled cell lines that reflect different cALL subtypes, as defined by diverse genomic rearrangements, a balanced mix of female and male donor patients and different B cell developmental stages of origin (Fig. 1a). In total, we identified 243,929 unique peptides mapping to 16,094 gene symbols across cell lines with similar global melting profiles (Fig. 1b and Supplementary Fig. 1). We obtained deep peptide coverage per gene symbol (Supplementary Fig. 2a) by measuring a total of 114 HiRIEF fractions per sample by liquid chromatography with tandem mass spectrometry (LC–MS/MS) analysis^18^.

**Fig. 2:**
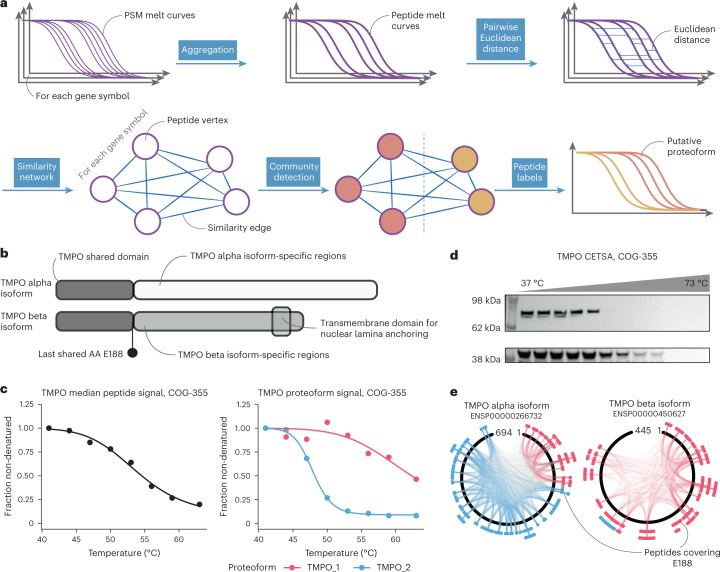
Proteoform detection based on peptide melting curve similarity. **a**, Schematic of the method. **b**, Schematic of the domains of the TMPO alpha and beta isoforms. **c**, Melting curves of the median TMPO peptide signal (left) and by proteoforms as detected by the above outlined approach. **d**, Western blot validation (*n* = 1) of the detected proteoforms for TMPO (alpha on top, beta below) with differential thermal stability in the COG-355 cell line. **e**, Mapping of peptides assigned to the different proteoforms to the alpha and beta isoforms of TMPO. Source data

As expected for proteoforms with different cellular functions, we observed that peptides mapping to a single gene symbol often formed groups with distinct thermal stability patterns. In fact, grouping of peptides by thermal stability reflected annotated proteoforms for individual proteins (illustrative examples in Supplementary Fig. 2b,c). We, thus, exploited clustering of similar peptide melting profiles by developing a method—pepnet—to assign peptides to different proteoforms without relying on their annotation (Fig. 2a). To do so, we filtered our dataset to contain only peptides that had been identified and quantified in at least two cell lines and computed pairwise similarities between all melting curves of peptides mapping to the same gene symbol. Then, for each gene symbol, a fully connected graph was constructed based on respective peptide similarities, and clusters were detected using the Leiden algorithm. We accepted all recovered clusters supported by at least three unique peptides and modularity *Q* > 1 × 10^−13^, which was found to control the false discovery rate (FDR) at 10% when evaluating our method on simulated datasets (Supplementary Fig. 3). This resulted in detection of 15,846 functional proteoform groups of 9,290 genes, with most genes being represented by one (44%) or two (44%) and a maximum of five proteoform groups (Supplementary Fig. 4a and Supplementary Data 1). As expected, our derived proteoforms showed higher modularity than Ensembl annotated ones (Supplementary Fig. 4b), suggesting that this approach extends delineation of proteoforms in comparison to existing annotations, with 23% of detected proteoform groups reflecting currently annotated proteoforms (illustrative examples in Supplementary Fig. 5a,b). Proteins with detected proteoform groups were analyzed in terms of different features, such as subcellular localization, length, protein abundance and half-life, and compared to proteins with known isoforms (Supplementary Fig. 6a–s). We found that globally similar trends were observed for these parameters between proteins with detected proteoform groups and those with annotated isoforms, except for protein abundance and length-normalized peptide coverage. When examining detected proteoforms in detail, we confirmed our approach by identifying proteoforms representing previously described cases of alternative splicing and proteolytic cleavage. For example, lamina-associated polypeptide 2 (TMPO) is a protein known to be expressed in several isoforms generated via alternative splicing. Two functionally important isoforms, alpha and beta, share a common N-terminus but differ in their C-termini^21^. The TMPO alpha isoform associates with chromatin in a cell-cycle-dependent manner, and TMPO beta isoform associates with the inner nuclear lamina via a transmembrane domain and facilitates lamin-mediated structural organization of chromatin (Fig. 2b)^22^. Using our proteoform detection method, we found two distinctly melting proteoforms for TMPO (Fig. 2c). We used an antibody recognizing the TMPO N-terminus to confirm differential melting for bands at molecular weights corresponding to alpha and beta isoforms (Fig. 2d). Furthermore, we observed that most peptides assigned to proteoform 1 (TMPO_1) were specifically mapping either to the sequence of the TMPO beta isoform or to the joint N-terminus of both isoforms (Fig. 2e). Thus, our method successfully detected the TMPO alpha and beta isoforms solely by considering the melting profiles of the peptides across cell lines mapping to the respective gene symbol.

**Fig. 3:**
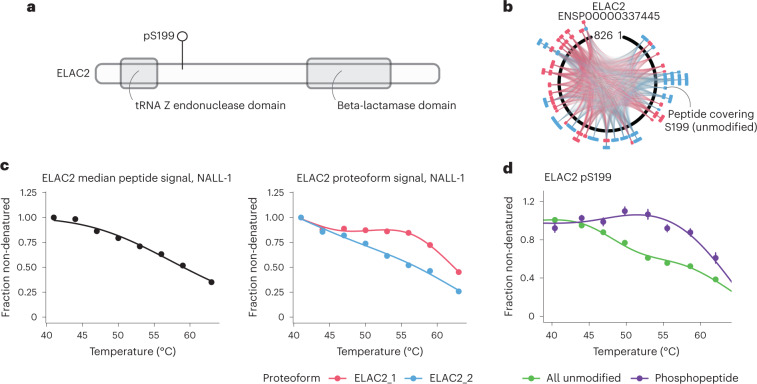
Proteoform detection example ELAC2. **a**, Schematic of the protein domains of ELAC2. **b**, Mapping of peptides assigned to the different ELAC2 proteoforms to the protein sequence. **c**, Melting curves of the median ELAC2 peptide signal (left) and by detected proteoforms. **d**, Melting curves of unmodified and pS199 phosphorylated ELAC2 in HeLa cells in ref. ^16^. Error bars represent s.e.m. Source data

In another example, we identified two proteoforms of the zinc phosphodiesterase ELAC2 (Fig. 3a,b), an enzyme known to localize to the nucleus and to mitochondria^23^. Although ELAC2_2, comprising an unmodified peptide covering serine 199 (S199), showed a profile similar to the median peptide signal per gene symbol, ELAC2_1 displayed a pattern (Fig. 3c) reminiscent of a differentially melting proteoform phosphorylated on S199 that we observed in a previous study using a phosphoTPP experiment (Fig. 3d)^16^. To corroborate ELAC2_1 as the pS199 phospho-proteoform of ELAC2, we queried our dataset against the human database, this time including phosphorylation as a modification. In fact, we found a peptide capturing the pS199 site of ELAC2 that showed a thermal stability pattern similar to ELAC2_1 and the pS199 phospho-proteoform identified in the phosphoTPP experiment (Supplementary Fig. 7). Therefore, our proteoform detection approach successfully identified post-translationally modified subpools of the same protein without the need for peptide enrichment.

In addition to these examples, we found several cases of proteoforms that resulted from proteolytic cleavage—for example pre-saposin (PSAP) (Supplementary Fig. 8a–d) and NOTCH1 (Supplementary Fig. 8e–g). These results are also in agreement with previous studies that established the existence and biological relevance of these proteoforms, further validating our approach.

Taken together, the peptide-level TPP data and the new proteoform detection algorithm allowed us to identify different proteoforms that reflected known functional characteristics of the respective proteins.

### Proteoform thermal stability reflects B cell biology

**Fig. 4:**
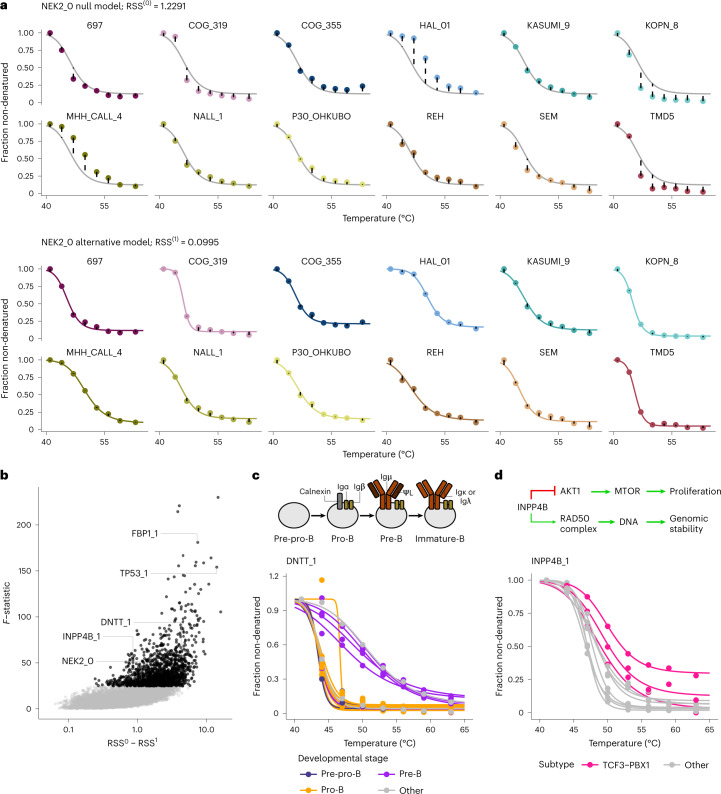
NPARC across cell lines. **a**, Exemplary fits of the null (top) and alternative (bottom) models to the cell line melting profiles of NEK2_0, the only proteoform found for NEK2. **b**, Volcano plot of the results obtained from the differential analysis. Black points represent proteoforms with the top 10% of *F*-statistics taken as differentially melting across cell lines (*F* ≥ *P*_90%_(F)). **c**, Melting profile of DNTT_1 across cell lines color-labeled by B cell developmental stage. **d**, Melting profile of INPP4B_1 across cell lines color-labeled by cell line genomic aberration subtype. Source data

Thermal stability of proteins can vary across cell lines, reflecting genomic variation, specific protein interaction networks and differential pathway activity^17^. To explore this aspect, we sought to identify differential thermally stable proteoforms across our samples. To do so, we performed non-parametric analysis of response curves (NPARC)^24^ to find differences across the 20 cALL cell lines (illustrated by NEK2 kinase peptide profiles in Fig. 4a). This allowed us to detect 1,408 proteoforms with differential melting curves (90% percentile of observed *F*-statistics, a heuristic based on the observed reproducible differences at this cutoff rather than a concrete error rate control that was not readily applicable here) across the profiled cell lines (Fig. 4b and Supplementary Data 2). A similar analysis at the protein level leads to a lower overall *F*-statistic per protein symbol (median = 8.30, compared to 9.17 when performed at the proteoform group level). This indicates that our grouping enables us to detect differences between some proteoforms that are hidden at the protein level. Among the top hits of the analysis, we found a proteoform of p53 (TP53_1), a tumor suppressor protein, and fructose-1,6-bisphosphatase 1 (FBP1_1), a rate-limiting enzyme of gluconeogenesis (Supplementary Fig. 9a). For the NPARC hits, we sought to identify potential mechanisms behind differential melting of these proteoforms and, therefore, annotate differences in proteoform functional roles in different cell line backgrounds. Although the differential thermal stability of TP53_1 could be related to altered protein interactions (see next section), in the case of FBP1_1 the higher thermal stability of the cluster of proteoform peptides was associated with high FBP1 protein abundance in respective cell lines (*P* = 3.3 × 10^−10^, two-sided Welch two-sample *t*-test on protein fold changes; Supplementary Fig. 9b). However, as we did not observe global correlation between thermal stability and abundance (Supplementary Fig. 9c), these data suggested a specific effect linked to higher FBP1 activity in these cell lines. Previous studies had shown that cell lines with high FBP1 abundance display activation of the pentose phosphate pathway, resulting in chemotherapy resistance and poor clinical outcome in acute myeloid leukemia^25^. In agreement with these observations, we found higher thermal stability of all proteoforms of glucose-6-phosphate dehydrogenase, the rate-limiting enzyme in the oxidative pentose phosphate pathway, in the cell lines with high thermal stability of FBP1_1, although not all of them were significant (Supplementary Fig. 9d). This illustrates how our data can be used to identify functional links based on the differential thermal stability of proteoforms.

Another protein with differential thermal stability across cell lines was the DNA nucleotidylexotransferase proteoform 1 (DNTT_1), a DNA polymerase that adds random nucleotides to the junction of rearranged immunoglobulin chains during B cell maturation^26^. We identified strikingly distinct melting profiles (Fig. 4c) that were associated (*P* = 0.036, Fisher test) with the B cell progenitor origin of the acute lymphoblastic leukemia cell lines^27^. DNTT diversifies the variable region of the Ig-light chain during the pre-B stage and diversifies Ig-heavy chain variable regions during the pro-B stage^28,29^. Thus, higher thermal stability may indicate differences in DNTT–DNA binding dynamics between the developmental stages.

We also found that INPP4B_1, a proteoform of INPP4B, a protein and lipid phosphatase that antagonizes the PI3K/Akt signaling pathway^30^, showed higher thermal stability in cell lines of the TCF3-PBX1 subtype (*P* = 0.017, two-sided Wilcoxon rank-sum test on area under the melting curves; Fig. 4d). INPP4B has also been shown to be involved in maintaining genomic integrity through associations with RAD50 in the nucleus, and loss of INPP4B was shown to sensitize cells to PARP inhibition^31^. We observed that TCF3–PBX1 fusion cells had decreased INPP4B abundance at baseline^27^ (*P* = 0.039, two-sided Welch two-sample *t*-test on protein fold changes) and were selectively sensitive to the PARP inhibitor talazoparib (*P* = 0.011, two-sided Welch two-sample *t*-test on selective drug sensitivity scores (sDSS)^27^). This suggests that this proteoform is associated with sensitivity to drug treatment that reduces genomic stability, which could implicate nuclear relocalization in the cell lines with observed high thermal stability.

Overall, we detected hundreds of examples of proteoforms with differential thermal stability in the cALL cell lines studied. Because thermal stability reflects the state and activity of proteins in a complementary way to traditional abundance proteomics^12,15^, these examples pinpoint pathway activation status and reveal new candidate biomarkers for therapy.

### Co-aggregation indicates differential proteoform interactions

**Fig. 5:**
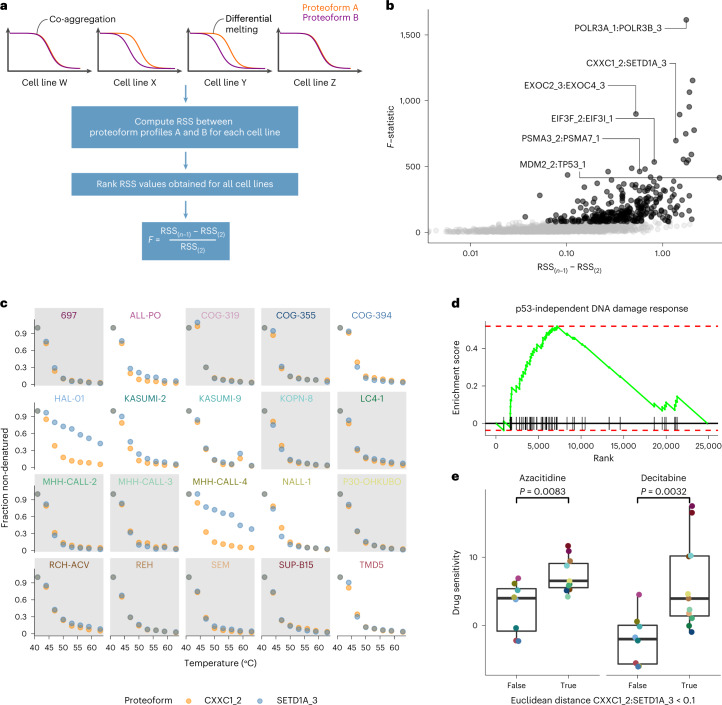
Differential proteoform co-aggregation analysis. **a**, Schematic of the performed analysis. To obtain robust results, the *F*-statistic is computed based on the difference of the second highest (RSS_(*n*−1)_) and second lowest (RSS_(2)_) residual sum of squares between the two proteoforms in all cell lines. **b**, Volcano plot of the results of the analysis. RSS_(*n*−1)_ − RSS_(2)_ represents the effect size—that is, the difference between the profiles of proteoform A and B in the cell line with the second highest and second lowest distance. **c**, Profiles of CXXC1_2 and SETD1A_3 across cell lines showing their co-aggregation in some cell lines (gray background) and differential melting (white background) in other cell lines. **d**, Enrichment plot for genes part of the ‘p53-Independent DNA Damage Response’ set based on differentially expressed transcripts between cell lines with co-aggregation of CXXC1_2 and SETD1A_3 versus all others (NES = 1.82; *P*_adj._ = 0.01, Kolmogorov–Smirnov test with Benjamini–Hochberg method for multiple testing adjustment). **e**, Box plots of drug sensitivity of cell lines with CXXC1_2 and SETD1A_3 co-aggregation (*n* = 11) versus all others (*n* = 8) to two different nucleoside analogs. The *P* values shown were obtained from a two-sided Welch two-sample *t*-test. Center lines in all box plots represent the median; the bounds of the boxes are the 75th and 25th percentiles—that is, the interquartile range; and the whiskers correspond to the highest or lowest respective value. Source data

Melting curves of interacting proteins (PPIs) or complex members have been shown to often coincide, a feature attributed to co-aggregation of the respective interactors^13^. Recently, we exploited this concept to test for differential co-aggregation of protein interactors between two conditions^32^. Here, we adapted this approach to a robust multigroup comparison (Fig. 5a) to detect differential proteoform–proteoform interactions (PFPFIs) across the profiled cALL cell lines using an extended PPI annotation of the STRING database^33^. Benchmarking the PPI prediction of deep TPP versus the size-exclusion chromatography coupled to mass spectrometry (SEC–MS) dataset^34^ showed a slightly inferior predictive power for our deep TPP approach (Supplementary Fig. 10). In total, we tested 2,901 PFPFIs, which showed co-aggregation in at least one of the cell lines, for differential co-aggregation across cell lines. We considered PFPFIs within the top 10% of obtained *F*-statistics (290 PFPFIs) as significantly differential across cell lines (Fig. 5b and Supplementary Data 3). Among those cases, we identified several examples of differential intra-complex PFPFIs, potentially reflecting varying degrees of complex assembly or activity across the profiled cell lines (Supplementary Fig. 11a–e).

One differentially co-aggregating proteoform pair was MDM2_2 and TP53_1 (Supplementary Fig. 11e). MDM2 is an E3 ubiquitin ligase that is known to ubiquitinate the tumor suppressor p53 and, thus, promote its degradation^35^. Furthermore, MDM2 is often upregulated in different cancers, leading to increased degradation of p53, resulting in uncontrolled cell division^36^. We, thus, wondered whether cell lines in which MDM2_2 and TP53_1 co-aggregated, which we interpreted as a sign of MDM2 binding to p53 and promoting its degradation, were more susceptible to MDM2 inhibition than other cell lines. Indeed, the two cell lines that featured co-aggregation of MDM2_2 and TP53_1, LC4-1 and P30-OHKUBO, showed higher sensitivity to idasanutlin, an MDM2 inhibitor, compared to other cell lines (*P* = 0.02, two-sided Welch two-sample *t*-test; Supplementary Fig. 9f). Additionally, when examining *TP53* and *MDM2* mutation status in the DepMap sequencing dataset^37^, these cell lines featuring co-aggregation of the two proteoforms did not have mutations in the respective proteins, whereas other cell lines included both mutated and unmutated genotypes. This showcases how our strategy can reveal functionally relevant connections between proteins and use them to generate hypotheses on drug sensitivity.

We also found the differentially co-aggregating proteoform pair CXXC1_2 and SETD1A_3 (Fig. 5c). SETD1A is a SET domain containing histone methyltransferase, which has been reported to mediate DNA damage response^38^, and CXXC1 was found to regulate SETD1A activity^39^. We hypothesized that co-aggregation of CXXC1_2 and SETD1A_3 could reflect an ongoing DNA damage response in respective cell lines. In fact, comparing RNA sequencing (RNA-seq) profiles^27^ of cell lines with co-aggregating versus differential CXXC1_2 and SETD1A_3 melting profiles revealed that the gene set ‘p53-Independent DNA Damage Response’ was significantly enriched among upregulated genes in cell lines that featured co-aggregation of this proteoform pair (Fig. 5d). We further asked whether these cell lines showed altered sensitivity to DNA damage-inducing drugs, such as nucleoside analogs. Consistent with this hypothesis, we observed significantly higher sensitivity to the nucleoside analogs and hypomethylating agents azacitidine and decitabine for cell lines in which CXXC1_2 and SETD1A_3 co-aggregated (Fig. 5e).

Taken together, we present an approach for the detection of differentially co-aggregating pairs of proteoforms and show that some of these altered interactions can be linked to activity of cellular processes and drug response.

### Proteoform thermal stabilities as drug response biomarkers

**Fig. 6:**
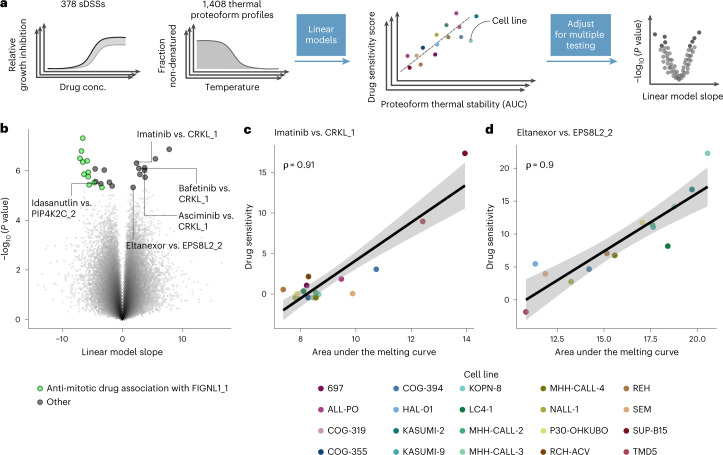
Association of thermal proteoform stability to drug sensitivity across cALL cell lines. **a**, Schematic of the strategy to test for thermal proteoform stability drug sensitivity associations. Linear models were built using limma to test for correlation between thermal stability and drug response; obtained *P* values were adjusted for multiple testing using the Benjamini–Hochberg method. AUC, area under the curve. **b**, Volcano plot representing the results obtained from the limma linear modeling workflow depicted in **a**. **c**,**d**, Scatter plot of CRKL_1 and EPS8L2_2 thermal stability and cell line drug sensitivity to imatinib and eltanexor, respectively. The Pearson correlation coefficient (⍴) is shown in each scatter plot. The linear regression trendline (black) and its 95% confidence interval (shaded gray area) are shown in the scatter plots. Source data

Encouraged by the observed associations between pathway activity (reflected in protein thermal stability) and drug sensitivity, we sought to generalize this principle across a larger drug panel—namely, the 528 drugs used in our previous study^27^. By using limma^40^ to correlate previously published sDSSs^27^ of 378 drugs with a minimal effect cutoff on any of the profiled cell lines (sDSS ≥ 6) with all previously determined 1,408 differentially thermally stable proteoforms (Fig. 6a), we retrieved 26 significant drug–proteoform thermal stability associations (*P*_*adj*._ *<* 0.1, Benjamini–Hochberg method) (Fig. 6b and Supplementary Data 4). Among these, we found thermal stability of CRKL_1 to be positively correlated with sensitivity to the BCR-ABL inhibitors imatinib, asciminib and bafetinib (Fig. 6b, c). CRKL is an adapter protein downstream of ABL1 that is phosphorylated upon activation of ABL1 (ref. ^41^). Previously, it was observed that CRKL was thermally destabilized upon treatment with dasatinib, another BCR-ABL inhibitor^8^. Inversely, thermal stabilization of CRKL appears to be related to active ABL1 signaling, which is in line with a positive correlation of sensitivity to BCR-ABL1 inhibitors (Fig. 6c and Supplementary Fig. 12). Moreover, we found that cell line sensitivity to several anti-mitotic drugs was negatively correlated with figetin-like protein (FIGNL1) proteoform 1 (FIGNL1_1) thermal stability (Fig. 6b and Supplementary Fig. 13a–f). FIGNL1 is involved in DNA double-strand repair via homologous recombination^42^. Because FIGNL1_1 thermal stability was negatively correlated with FIGNL1 protein abundance (⍴ = −0.64, *P* = 0.0025), high FIGNL1_1 thermal stability could reflect active engagement in the FIGNL1-containing complex to resolve DNA double-strand breaks. Indeed, correlation of FIGNL1_1 thermal stability with anti-mitotic drug sensitivity (⍴ = −0.9 and ⍴ = −0.86 for eribulin and vinorelbine, respectively) was stronger than for FIGNL1 abundance (⍴ = −0.7 and ⍴ = −0.68 for eribulin and vinorelbine, respectively). Furthermore, the proteoform FIGLN1_2 did not correlate significantly with drug sensitivity (Supplementary Fig. 13g–i), suggesting a specific role for FIGLN1_1. Thus, high activity of the FIGNL1_1-containing complex could lead to reduced mitotic exit at cell cycle checkpoints and may, thus, explain lower sensitivity to anti-mitotic drugs.

Another interesting hit was the positive correlation of PIP4K2C_2 thermal stability with cell line sensitivity to the MDM2 inhibitor idasanutlin (Supplementary Fig. 14a,b). Several PIP4K2 family members were previously linked to promotion of tumorigenesis in the context of p53 loss of function^43^. Thus, it appears plausible that high thermal stability of PIP4K2C_2, potentially reflecting a higher fraction of cofactor-bound protein pool, is associated with increased sensitivity to MDM2 inhibition, as the related signaling pathway appears to lead only to cell growth in the absence of p53 function. The fact that the correlation of idasanutlin sensitivity to PIP4K2C abundance is weaker and positive rather than negative (Supplementary Fig. 14c), and that stability of other PIP4K2C proteoforms is also strongly correlated to drug response (Supplementary Fig. 14d–f), reinforces the notion that thermal stability gives a more functional readout of protein state than measurements of protein abundance.

Finally, we detected a positive correlation between EPS8L2_2 (epidermal growth factor receptor kinase substrate 8-like protein 2) with eltanexor (Fig. 6c), a nuclear export inhibitor. EPS8L2 is known to form a complex with SOS1 and ABI1, which is involved in regulating actin remodeling^44^. The observed correlation was specific to thermal stability and to the EPS8L2_2 proteoform (Supplementary Fig. 15a–d). To investigate how high EPS8L2_2 thermal stability could confer sensitivity to nuclear export inhibition, we performed a differential expression analysis between cell lines with high and low EPS8L2_2 thermal stability. When performing Gene Ontology (GO) molecular function enrichment analysis on the transcripts upregulated in cell lines with high EPS8L2_2 thermal stability, we found a significant enrichment (*P*_adj._ < 0.1) of the terms ‘actin binding’, ‘antigen binding’ and ‘immunoglobulin receptor binding’. This may indicate that high EPS8L2_2 could reflect actin remodeling in response to B cell receptor (BCR) activation^45^. It was shown previously that nuclear export inhibition suppresses downstream effects of BCR signaling in chronic lymphocytic leukemia^46^; therefore, it is plausible that eltanexor treatment may be effective in a subset of acute lymphoblastic leukemias relying on BCR signaling for proliferation.

## Discussion

CETSA and TPP were developed with the primary goal of detecting protein targets of drugs^8,10^. However, it has been realized that these methods can also detect other sources of protein biophysical variation that are difficult to quantify with other proteomics methods, including protein interactions with other biomolecules^47^. Since the adaptation of the method to infer functional phosphorylation sites^16^, it has also become clear that TPP bears the potential for detecting post-translationally modified proteoforms. In the present study, we performed TPP with unprecedented peptide coverage and generalized this concept to enable unbiased detection of co-existing functional proteoform groups. The detected events of diversified protein products comprise cases of alternative splicing, proteolytic cleavage, post-translational modifications and variants interacting with metabolites, proteins or DNA.

Previous efforts used linear models^4^ or peptide correlation combined with hierarchical clustering for detection of functional proteoform groups from bottom-up proteomics datasets, such as full proteome or size-exclusion experiments. However, underlying sample conditions need to reveal proteoform differences at the level of protein abundance for these methods to work. Furthermore, analysis methods either have been designed to specifically detect single outlier peptides, in the case of PeCorA, or require specification of the number of proteoforms expected per protein, in the case of the COPF algorithm^5^. Our approach, instead, measures peptide thermal stability, which can reflect any form of thermodynamic perturbation of a protein’s makeup or its interactions. This has been shown to reveal various types of functional proteoforms, such as fusion events^8^, post-translational modifications^16^, differential^12^ PPIs and spliced and proteolytically cleaved isoforms. Combining this readout across different cell lines further increases robustness of the approach toward outlier peptides and sensitivity to detect subtle differences between proteoforms. Our FDR-controlled graph-based analysis strategy is able to flexibly decide how many functional proteoform groups a protein has (from a single proteoform to any number). It makes use of peptide profile similarity rather than correlation, the latter being problematic in the context of TPP due to the high inherent correlation of peptide melting curves. One of the limitations of our strategy is that it relies on identifying a minimal number of peptides per protein to detect proteoforms. Because longer proteins give rise to more tryptic peptides, we are more likely to find proteoforms for longer proteins (Supplementary Fig. 6). However, longer proteins are also known to have more isoforms (Supplementary Fig. 6). Additionally, we require different proteoforms of a protein to differ in thermal stability by a certain temperature—with the analysis of our simulated benchmark dataset revealing that the sensitivity of our method for detection of proteoforms differing in melting temperature by less than 4 °C depends on the peptide coverage and the intra-proteoform peptide noise level (Supplementary Fig. 3c–e). We apply a filter for a minimum of three peptides supporting a functional proteoform group as a tradeoff between allowing detection of proteoforms with few supporting peptides but reducing false positives supported only by outlier peptides or missed cleavage peptides that share high sequence identity. Moreover, the predictive power of deep TPP for inference of annotated PPIs, although informative on interactions present in live cells, is slightly inferior to SEC–MS when considering the same number of observations (Supplementary Fig. 10). Lastly, the TMT multiplexed measurement of eight deeply fractionated different heat treatment conditions in two cell lines requires considerable MS time (~6 days); however, with the constant advancements of more sensitive and faster instruments, we think that this method will become amenable to even more researchers in the future and possibly, at some point, albeit in a more targeted fashion, for the analysis of clinical samples. To enable cross-disciplinary engagement with our results, we created an interactive web application: https://www.proteomics.se/deepmeltome/.

In conclusion, we show that performing TPP with high peptide coverage allows for the detection of proteoform groups and simultaneous inference of functional aspects by revealing peptide sequence coverage, differences in PPIs and associations with drug response. By integrating thermal stability of proteoforms, transcriptomics and drug sensitivity profiling data across cell lines, we demonstrate that it is possible to identify biomarkers for cellular processes and drug response. Thus, we think that deep TPP for proteoform detection is a broadly applicable and complementary addition to existing technologies for delineating proteoforms and for supporting analytical strategies interrogating proteoform composition and contribution to cellular processes.

## Methods

### Cell cultivation

The 20 childhood B cell precursor acute lymphoblastic leukemia (BCP-ALL) cell lines used in this study were obtained from the Deutsche Sammlung von Mikroorganismen und Zellkulturen (DSMZ, German Collection of Microorganisms and Cell Cultures), the Children’s Oncology Group^48^ Childhood Cancer Repository, the American Type Culture Collection, the Japanese Collection of Research Bioresources Cell Bank, the European Collection of Authenticated Cell Cultures and the Banca Biologica e Cell Factory. RPMI 1640 (AQmedia, Sigma-Aldrich) or IMDM (Sigma-Aldrich) supplemented with either 10% or 20% FBS (Sigma-Aldrich), 20 mM HEPES (Gibco/Life Technologies), 1 mM sodium pyruvate (Sigma-Aldrich), 1× MEM non-essential amino acids (Sigma-Aldrich) and 1× penicillin–streptomycin (Sigma-Aldrich) was preferably used. Cell line provider details, culture conditions and growth media are also described in Supplementary Table 1 and in our previous study^27^. Cell lines were grown at 37 °C and 5% CO_2_ to a cell density of approximately 1–2 million cells per milliliter. Cells were harvested at 500*g* for 3 minutes and washed twice with HBSS (Gibco; no calcium, no magnesium and no phenol red).

### Sample preparation for LC–MS/MS

#### TPP of the cell lines

Freshly washed cells were resuspended to a density of 100 million cells per milliliter in HBSS and distributed as aliquots of 10 million cells into eight 0.2-ml PCR tubes. Tubes were heated in parallel for 3 minutes to 41, 44, 47, 50, 53, 56, 59 and 63 °C, followed by a 3-minute incubation time at room temperature. Afterwards, cells were flash-frozen in liquid nitrogen.

### Digest and TMT labeling

Lysis was performed by five freeze–thaw cycles using a 25 °C heating block and liquid nitrogen. Cell debris and precipitated proteins were removed by centrifugation at 21,000*g* and 4 °C for 40 minutes. Supernatants were transferred to new tubes, and protein concentrations were determined using the DC protein assay according to standard protocols provided by the kit manufacturer (Bio-Rad). Equal volumes of soluble protein supernatants were transferred to new tubes and subjected to in-solution digestion. First, the samples were supplemented with reagents to contain a final concentration of 50 mM TEAB, 0.1% SDS and 5 mM TCEP. Reduction was performed at 65 °C for 30 minutes. Samples were then cooled down to room temperature and alkylated with 15 mM of chloroacetamide for 30 minutes. Proteins were digested overnight with 1:40 Lys-C (Wako Chemicals)-to-protein ratio. Consecutively, trypsin (Thermo Fisher Scientific) was added at a 1:70 enzyme-to-protein ratio for an 8-hour incubation at 37 °C. Finally, the same amount of trypsin was added one more time for an overnight incubation. Resulting peptides were labeled by 16-plex TMTpro tags (TMTpro, Thermo Fisher Scientific) using the same amount of respective label for each sample. Eight melting points of two randomly selected cell lines were combined in each TMT 16-plex set. The protein amounts were adjusted to contain the same total protein amount for all cell lines throughout the TMT sets. An overview of the sets is given in Supplementary Table 1. Labeling was performed according to the manufacturer’s instructions but with 2-hour incubation before quenching the TMT labeling reaction. Labeling efficiency was determined by LC–MS/MS before mixing the TMT-labeled samples. Sample cleanup was performed using solid-phase extraction Strata-X-C SPE columns (Phenomenex). Purified peptides were dried in a vacuum centrifuge.

### HiRIEF of peptides

The pre-fractionation method was applied as previously described^18^. Sample pools of ~300 µg were subjected to peptide IEF-IPG (isoelectric focusing by immobilized pH gradient) in a pH range of 3–10 and 3.7–4.9, respectively. Dried peptide samples were dissolved in 250 µl of rehydration solution of 8 M urea containing 1% IPG pharmalyte pH 3–10 or 2.5–5, respectively (GE Healthcare) and allowed to adsorb to the gel bridge strip and the 24-cm linear gradient IPG strips (GE Healthcare) by swelling overnight. After focusing, the peptides were passively eluted into 72 contiguous fractions with MilliQ water/35% acetonitrile (ACN)/35% ACN + 0.1% formic acid (FA) using an in-house-constructed IPG extraction robot (GE Healthcare Bio-Sciences AB, prototype instrument) into a 96-well plate (V-bottom, Greiner, 651201), which were then dried in a SpeedVac. The resulting fractions were dried and kept at −20 °C.

### LC–MS/MS runs of the HiRIEF fractions

Online LC–MS was performed using a Dionex UltiMate 3000 RSLCnano System coupled to a Q-Exactive HF mass spectrometer (Thermo Fisher Scientific). Each fraction was subjected to MS analysis. Samples were trapped on a C18 guard-desalting column (Acclaim PepMap 100, 75 μm × 2 cm, nanoViper, C18, 5 µm, 100 Å) and separated on a 50-cm-long C18 column (EASY-Spray PepMap RSLC, C18, 2 μm, 100 Å, 75 μm × 50 cm). The nano capillary solvent A was 95% water, 5% DMSO and 0.1% FA; solvent B was 5% water, 5% DMSO, 95% ACN and 0.1% FA. At a constant flow of 0.25 μl min^−1^, the curved gradient went from 2% B up to 40% B in each fraction, as shown in Supplementary Data 5, followed by a steep increase to 100% B in 5 minutes. FTMS master scans with 60,000 resolution (and mass range 300–1,500 *m*/*z*) were followed by data-dependent MS/MS (35,000 resolution) on the top five ions using higher-energy collision dissociation at 30% normalized collision energy. Precursors were isolated with a 1.2-*m*/*z* window. Automatic gain control targets were 1 × 10^6^ for MS1 and 1 × 10^5^ for MS2. Maximum injection times were 100 ms for MS1 and 100 ms for MS2. Dynamic exclusion was set to 30-second duration. Precursors with unassigned charge state or charge state 1 were excluded. An underfill ratio of 1% was used.

### Analysis of LC–MS/MS runs

Orbitrap raw MS/MS files were converted to mzML format using msConvert from the ProteoWizard tool suite^49^. Spectra were then searched using MSGF+ (v10072)^50^ and Percolator (version 2.08)^51^, where search results from eight subsequent fractions were grouped for Percolator target/decoy analysis. All searches were done against the human protein subset of Ensembl 99 in the Galaxy platform^52^. MS-GF+ settings included precursor mass tolerance of 10 ppm, fully tryptic peptides, maximum peptide length of 50 amino acids and a maximum charge of 6. Fixed modifications were TMTpro 16-plex on lysines and peptide N-termini and carbamidomethylation on cysteine residues, and a variable modification was used for oxidation on methionine residues. Quantification of TMTpro 16-plex reporter ions was done using IsobaricAnalyzer (version 2.0) of the OpenMS project^53^. Peptide-spectrum matches (PSMs) found at 1% FDR were used to infer gene identities. Protein quantification by TMTpro 16-plex reporter ions was calculated using TMT PSM ratios. The median PSM TMT reporter ratio from peptides unique to a gene symbol was used for quantification. Protein FDRs were calculated using the picked FDR method using gene symbols as protein groups and limited to 1% FDR^54^.

### Data pre-processing and proteoform detection

Quantitative reporter ion signal for PSMs was summarized on peptide level by summation. Reporter ion signals of all individual temperatures were normalized using variance stabilizing normalization^55^ and converted to fold changes relative to the first temperature. Next, to assign similarly melting peptides found to map to a certain gene symbol, a graph for each gene symbol was created connecting all peptides (vertices) with weights (edges) corresponding to their similarity in melting profile. The similarity was computed with

1 $$S_{ij} = \frac{1}{{1 + d_{ij}}}.$$

where *d*_*ij*_ is the weighted Euclidean distance between two peptides across all cell lines:

2 $$d_{ij} = \sqrt {\mathop {\sum }\limits_{n = 1}^N } \mathop {\sum}\limits_{k = 1}^K {\left( {x_i^{nk} - x_j^{nk}} \right)^2} \cdot \frac{v}{{NK}},$$

where $x_{\frac{i}{j}}^{nk}$ represents the fold change of peptide and respectively in cell line *n* at temperature *k*, and *v* represents the number of valid comparisons—that is, $\frac{v}{{NK}}$ is the fraction of fold changes without missing values of either peptide. Obtained graphs were then used for community detection using the Leiden algorithm^56^; however, only gene symbols for which at least ten peptides were identified and with at least two peptides per sample were subjected to this analysis (a detected community had to be supported by at least three peptides to be accepted to ensure that outlier peptides did not affect robust proteoform identification). Peptides mapping to gene symbols for which these criteria were not fulfilled were grouped to single proteoforms, and peptides mapping to gene symbols that were included in the community detection were assigned to proteoform groups if the modularity of the detected communities was higher than 1 × 10^−13^ and the peptide ambiguity ratio was lower than 0.5 (for peptides mapping to multiple genes, it is calculated as the number of ambiguous peptides divided by the sum of the number of gene-specific and ambiguous peptides). Modularity was computed using the function modularity() of the igraph R package. Through the assignment of peptides to communities, functional proteoform groups for each gene symbol were created. Summarization on proteoform group level was performed by summation of non-normalized raw peptide data assigned to individual communities. Obtained proteoform signal intensities were then normalized per temperature using variance stabilizing normalization, and relative fold changes to the lowest measured temperature were formed.

### Differential melting curve analysis

All functional proteoform groups detected in at least ten cell lines were fitted by a sigmoid function for each cell line individually. The sigmoid was fit using the NPARC R package implementation, which is defined as

3 $$f\left( T \right) = \frac{{1 - p}}{{1 + exp\left( {b - \frac{a}{T}} \right)}} + p{{{\mathrm{,}}}}$$

where *T* represents the temperature; *p* represents the plateau; and *a* and *b* are parameters affecting the slope and inflection point of the curve^8,24^. Fits for individual cell lines (alternative model for the NPARC method) were accepted if they had a residual standard deviation of *S*_*res*_ < 0.1 (for example, Supplementary Fig. 16), because high residuals due to a single cell line could hinder the detection of differential melting profiles in other cell lines. The residual sum of squares (RSS) was computed across cell lines as RSS^(1)^${{{\mathrm{RSS}}}}^{(0)} = \mathop {\sum}\nolimits_{n = 1}^N {\mathop {\sum}\nolimits_{k = 1}^K {\left( {f\left( {T_{nk}} \right)^{\left( 1 \right)} - x_{nk}} \right)^2} }$ and melting points (*f*(*T*_*m*_) = 0.5), and areas under the melting curve were computed for accepted fits of cell-line-specific proteoform thermal profiles by integration of the fitted sigmoid formulas. Null models were fit using the same sigmoid model (4) for each proteoform across all cell lines for which an alternative model fit was accepted. The null model RSS was computed as ${{{\mathrm{RSS}}}}^{(0)} = \mathop {\sum}\nolimits_{n = 1}^N {\mathop {\sum}\nolimits_{k = 1}^K {\left( {f\left( {T_{nk}} \right)^{\left( 0 \right)} - x_{nk}} \right)^2} }$. Based on the RSS of both models, an *F*-statistic was computed with

4 $$F = \frac{\mathrm{RSS^{\left( 0 \right)} - RSS^{\left( 1 \right)}}}{\mathrm{RSS^{\left( 0 \right)}}} \cdot \frac{{d_2}}{{d_1}},$$

where the degrees of freedom $d_1 = \upsilon _1 - \upsilon _0$ and $d_2 = p_i-\upsilon _0$, with *p*_*i*_, *υ*_0_ and *υ*_1_ representing the number of observations for protein *i* and the number of parameters of the null and alternative model, respectively^24^. Proteoforms with an *F*-statistic above the 90th percentile were considered to have differential melting across cell lines. The rationale of considering this threshold for considering proteoforms differentially thermally stable across cell lines is the observation that, due to the heterogeneity across cell lines, most proteoforms do not reflect the expected distribution under the null hypothesis. However, this is assumed by the NPARC approach, which uses an empirical null model to infer significant deviance from the null. Although not controlling FDR at a fixed threshold, we chose this threshold, because proteoform groups with an *F*-statistic above the 90th percentile were found to have visually distinct thermal stability differences across cell lines.

### Differential proteoform–proteoform co-aggregation analysis

To test for pairs of proteoforms that co-aggregated in some cell lines but melted differentially in others, we adapted our previous approach for testing this between two conditions^32^. We started by extending the list of highly confident string interactions (combined score ≥ 950) by all possible proteoform interactions—that is, if protein A was previously annotated to interact with protein B and we detected three proteoforms for protein A and two for B, we replaced this entry by all possible 3 × 2 combinations. Next, we tested for co-aggregation of pairs of proteoforms in all individual cell lines using the approach described in ref. ^13^. All pairs of proteoforms that showed significant co-aggregation (*P*_adj._ < 0.1) in at least one of the cell lines were included for the differential analysis across cell lines. The test statistic for differences in co-aggregation across cell lines was determined by computing ${{{\mathrm{RSS}}}}_n = \mathop {\sum}\nolimits_{k = 1}^K {(x_k^A - x_k^B)^2}$ across all temperatures *k*, between all annotated pairs of proteoforms *A* and *B* for all individual cell lines *n*, ranking all RSS_*n*_ and computing

5 $$F = \frac{{\mathrm{RSS}_{(n - 1)} - \mathrm{RSS}_{(2)}}}{\mathrm{RSS_{(2)}}}.$$

Above, RSS_(__*n*__−__1__)_ and RSS_(__2__)_ represent the second highest and the second lowest RSS. In this way, the *F*-statistic became large only for cases in which at least two cell lines featured small and big differences between the melting curves of the two proteoforms, respectively. We considered *F*-statistics higher than the 90th percentile for further inspection due to difficult tractability of the underlying null distribution required to calibrate the *F*-statistic in terms of FDR.

### Proteoform thermal stability and drug response correlation

Proteoform thermal stabilities were associated with sDSSs by performing correlation analyses between the area under the melting curves of proteoforms found to differ across cell lines (90th percentile of observed *F*-statistics obtained from the NPARC analysis) with the sDSSs of the respective cell lines for all drugs with a minimal effect (sDSS < 6 for at least one cell line) using the R package limma^40^. Results obtained for all proteoforms and drugs were jointly adjusted for multiple testing using the Benjamini–Hochberg method^57^. Proteoform–sDSS associations with an adjusted *P* value of less than 0.1 were considered significant. The cell line COG-319 was excluded from the analysis because the sDSS for the cell line was an outlier that appeared unspecifically sensitive to most drugs, which negatively affected interpretation of drug sensitivity correlation results.

### Benchmark of the functional proteoform group detection method

To benchmark the PepNet algorithm for detection of proteoforms, we simulated two different datasets: (1) a dataset in which we generated 15 peptides per protein and (2) one with 50 peptides per protein. For each dataset, we simulated 1,000 negative proteins (that is, with no evidence for proteoforms) with varying melting points (ranging between 50 °C and 60 °C) with peptides that only differed by noise on two levels: (1) a melting point variability with standard deviation of 2 °C and (2) variability of the measured fold changes with standard deviation of 0.1, whereas variations below 0 were forbidden by forcing such cases to a small non-zero value. Additionally, we simulated a total of 200 positive proteins (that is, with evidence for two different proteoforms, with 50 proteins each differing by 1 °C, 2 °C, 3 °C and 4 °C, respectively) using the same sources of noise as for the negatives. For each peptide, we simulated eight-fold changes in 20 different cell lines, similar to our measurements in our true dataset.

We applied the PepNet algorithm to this dataset and sorted the results by modularity to perform a receiver operating characteristic (ROC) analysis checking whether true-positive proteoforms were ranked higher than true-negative ones.

To apply the COPF algorithm^5^ to the same dataset for comparison, we multiplied simulated fold changes by a factor of 1,000 because the algorithm expects intensity values rather than fold changes. Obtained results were ranked by the algorithm’s ‘proteoform_score’ and subjected to ROC analysis.

For both methods, proteoforms were accepted as correctly detected if two proteoforms were detected when simulated regardless of whether all peptides were correctly assigned to both IDs.

### Benchmark of PPI recapitulation

To benchmark the capability of deep TPP data to predict PPIs, we computed the average Euclidean distances between all pairs of quantified proteins as suggested previously^13^. We ranked protein pairs by increasing average distance and performed a ROC analysis using PPIs annotated by StringDB^33^ with a combined score of 900 or higher and direct and indirect PPIs within human protein complexes as annotated in ref. ^58^ as positives and all non-annotated PPI pairs as negatives.

To compare the capability of SEC–MS data to predict PPIs, we downloaded the interphase dataset in ref. ^34^ measured in HeLa cells. Intensity values measured in the different fractions were converted to fold changes by dividing by the highest value per protein across fractions. The dataset, which comprised 42 fractions, was then downsampled to eight fractions to have a similar number of observations per protein to the TPP dataset. We then employed the same procedure as for the deep TPP dataset to compute average Euclidean distances between pairs of proteins. The results were also sorted by increasing distances, and the same annotation of positives and negatives was used for ROC analysis.

### Differential RNA-seq analysis

Differential RNA-seq analysis was performed using DESeq2 (ref. ^59^). The sex of the cell line donors was included as a covariate in the design formula, when testing for a difference in conditions.

### Gene set enrichment analysis

Gene set enrichment analysis was performed using the log fold changes computed between the conditions of all genes using the R Bioconductor package fgsea.

### GO enrichment

GO enrichment was performed using the R Bioconductor package clusterProfiler^60^.

### CETSA temperature range analysis

COG-355 and ALL-PO cell suspensions were centrifuged at 300*g* for 5 minutes; the supernatant media was discarded; and the cells were washed twice with HBSS (Gibco/Life Technologies). Pelleted cells were resuspended in HBSS, and 75-µl cell suspensions (10 million cells) were aliquoted to 0.2-ml tubes. Samples were then heated in a temperature range of 37–70 °C in a Veriti Thermal Cycler (Applied Biosystems/Thermo Fisher Scientific) for 3 minutes, followed by 3-minute cooling at room temperature and immediate snap-freezing in liquid nitrogen. The cells were then lysed by three repeated freeze–thaw cycles and centrifuged at 21,000*g* for 40 minutes at 4 °C. The cleared supernatants were transferred to new tubes, denatured in LDS sample buffer (Thermo Fisher Scientific) and analyzed by western blotting.

### Western blotting

Cleared protein supernatants were denatured in LDS sample buffer (Thermo Fisher Scientific), resolved by SDS–PAGE using NuPAGE 4–12%, Bis-Tris gel (Invitrogen, Thermo Fisher Scientific) and NuPAGE MES SDS Running Buffer (Invitrogen, Thermo Fisher Scientific) and transferred to nitrocellulose membranes (Invitrogen, Thermo Fisher Scientific). SeeBlue Plus2 Pre-stained Standard was used as protein ladder (Invitrogen, Thermo Fisher Scientific). Afterwards, the membranes were blocked with 5% non-fat dry milk in TBST (Thermo Fisher Scientific) and incubated with primary antibodies for the appropriate target. TMPO/LAP2 (Thermo Fisher Scientific, A304-838A-M, RRID: AB_2782213 and PA5-96154, RRID:AB_2807956, 1:1,000 dilution), PSAP (Thermo Fisher Scientific, PA5-21340, 1:1,000 dilution, RRID: AB_11154619) and Saposin-C (Santa Cruz Biotechnology, sc-374119, 1:500 dilution, RRID: AB_10947406) antibodies were used for western blotting to detect corresponding targets. After overnight primary incubation at 4 °C, blots were rinsed using TBST and incubated with the appropriate HRP-conjugated secondary antibodies (Millipore, AP127P, RRID: AB_92472 for mouse primary antibody and Santa Cruz Biotechnology, sc-2004, RRID: AB_631746 for rabbit primary antibody, both used at a dilution of 1:5,000). All antibody incubations were diluted in 5% non-fat dry milk in TBST. Protein bands were developed with Clarity ECL Substrate Chemiluminescent HRP substrate (Bio-Rad) in a iBright CL1000 Imaging System (Invitrogen, Thermo Fisher Scientific). Bands were quantified using iBright Analysis Software version 4.0.1 (Thermo Fisher Scientific). Images of the full uncropped blots are provided with annotation in Supplementary Fig. 17 and Source Data.

### Reporting Summary

Further information on research design is available in the Nature Portfolio Reporting Summary linked to this article.

## Online content

Any methods, additional references, Nature Portfolio reporting summaries, source data, extended data, supplementary information, acknowledgements, peer review information; details of author contributions and competing interests; and statements of data and code availability are available at 10.1038/s41589-023-01284-8.

## Supplementary information

Supplementary Information Supplementary Table 1, Figs. 1–17 and References.

Reporting Summary

Supplementary Data 1 Table of detected proteoforms.

Supplementary Data 2 Differential melting from the NPARC analysis.

Supplementary Data 3 Table of PPI analysis.

Supplementary Data 4 Table of drug sensitivity–proteoform correlations.

Supplementary Data 5 HiRIEF gradient lengths.

## Data Availability

All proteomics datasets generated in this study have been deposited in PRIDE with the dataset identifier PXD031162. Annotations of proteins were based on the Ensembl 99, GRCh38.p13 human genome assembly, released on 16 January 2020. The post-search files and source data for supplementary figures were uploaded to Mendeley Data under DOI 10.17632/dwhtwh4dj7.2. The quantitative protein abundance data were taken from the PRIDE repository with the dataset identifier PXD023662. The RNA-seq data were taken from the National Center for Biotechnology Information’s Gene Expression Omnibus with accession number GSE168386. Analyzed data can be browsed using our interactive shiny app: https://www.proteomics.se/deepmeltome/. Source data are provided with this paper.

## Source data

Source Data Fig. 1 Statistical source data.
Source Data Fig. 2 Statistical source data, unprocessed western blots.
Source Data Fig. 3 Statistical source data.
Source Data Fig. 4 Statistical source data.
Source Data Fig. 5 Statistical source data.
Source Data Fig. 6 Statistical source data.

## Code availability

All code used to perform the computational analyses described and to reproduce the figures is available at https://github.com/nkurzaw/deepPedAllMeltome.
