# Supplementary material for: Deep thermal profiling for detection of functional proteoform groups
Source: Nat Chem Biol. 2023 Mar 20;19(8):962–71. doi: 10.1038/s41589-023-01284-8 (PMC10374440; doi:10.1038/s41589-023-01284-8)
Supplement: Supplementary file 1 — Supplementary Table 1, Figs. 1–17 and References. [file 41589_2023_1284_MOESM1_ESM.pdf]

nature chemical biology

Article

https://doi.org/10.1038/s41589-023-01284-8

Deep thermal profiling for detection of functional proteoform groups

In the format provided by the authors and unedited

Supplementary Table 1. TMT-set annotation and cell line information

tmt_set	labels	sample_name	subtype	sex	developmental_stage
Set1	126-130N	RCH-ACV	TCF3-PBX1	F	pro-B
Set1	134-130C	LC4-1	MEF2D-HNRNPUL1	F	early pre-B
Set2	126-130N	REH	ETV6-RUNX1	F	pre-pro-B
Set2	134-130C	P30-OHKUBO	MEF2D-HNRNPUL1	F	early pre-B
Set3	126-130N	KASUMI-9	MEF2D-HNRNPUL1	M	early pre-B
Set3	134-130C	SEM	KMT2A-AFF1	F	pre-B
Set4	134-130C	697	TCF3-PBX1	M	pro-B
Set4	126-130N	COG-355	ETV6-RUNX1	M	pro-B
Set5	134-130C	ALL-PO	KMT2A-AFF1	F	pre-pro-B
Set5	126-130N	KASUMI-2	TCF3-PBX1	M	pro-B
Set6	126-130N	SUP-B15	BCR-ABL1	M	pro-B
Set6	134-130C	MHH-CALL-3	TCF3-PBX1	F	pro-B
Set7	126-130N	TMD5	BCR-ABL1	M	pre-B
Set7	134-130C	KOPN-8	KMT2A-MLLT1	F	late pre-B
Set8	126-130N	NALL-1	PAX5-ETV6	M	pro-B
Set8	134-130C	COG-319	TCF3-PBX1	F	pro-B
Set9	126-130N	MHH-CALL-2	other	F	early pre-B
Set9	134-130C	COG-394	ZMIZ1-ABL1	F	late pre-B
Set10	126-130N	HAL-01	TCF3-HLF	F	pro-B
Set10	134-130C	MHH-CALL-4	IGH-CRLF2	M	pro-B
Set11	126-130N	RCH-ACV-BR2	TCF3-PBX1	F	pro-B
Set11	134-130C	MHH-CALL-3-BR2	TCF3-PBX1	F	pro-B

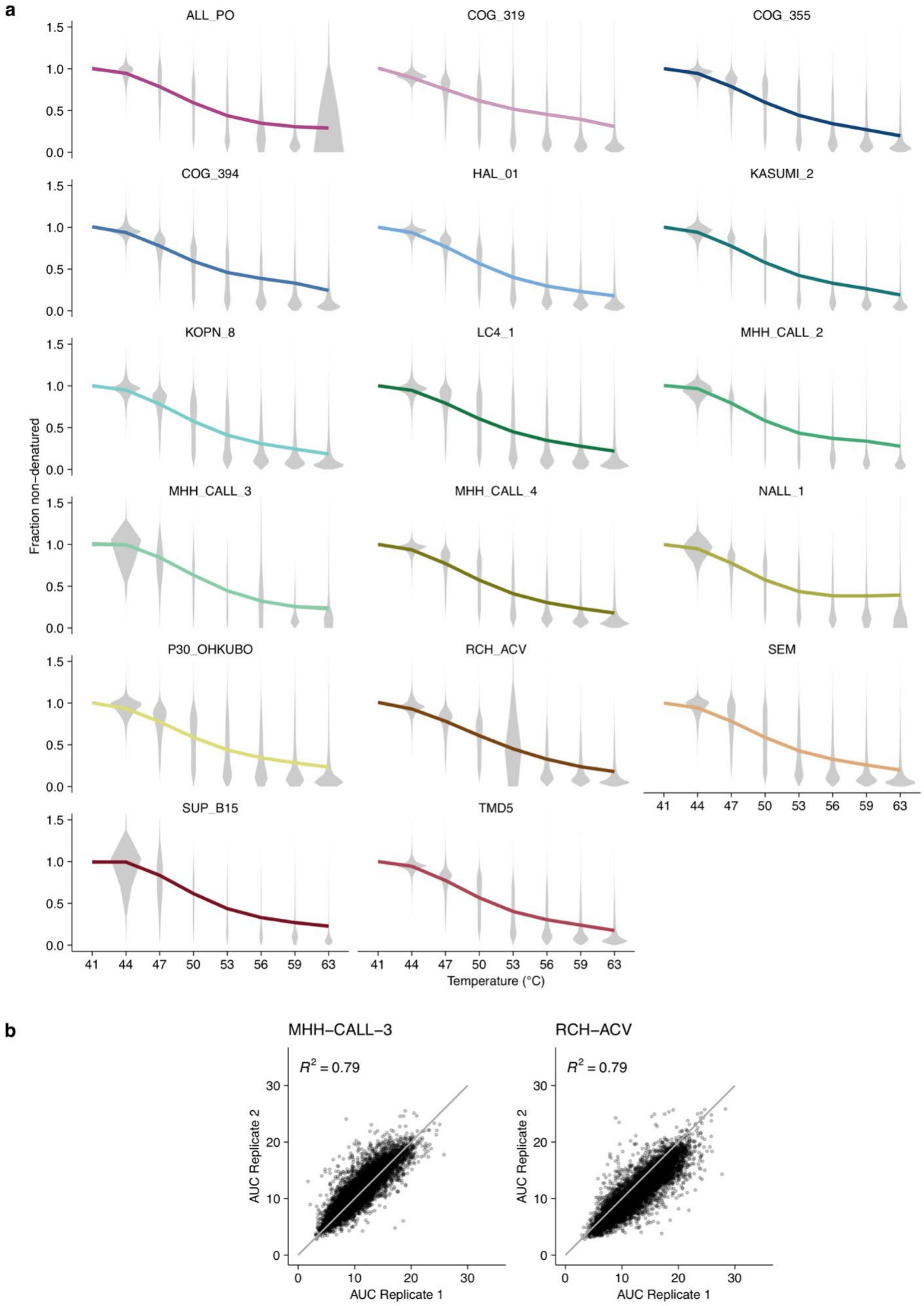

Supplementary Figure 1: **Global melting profiles of cell lines.** a) Average melting profiles after normalization across all peptides identified and quantified in all cell lines not shown in the main. b) Scatterplots of area under the melting curves (AUC) obtained from two biological replicates of detected proteoform groups in MHH-CALL-3 and RCH-ACV.

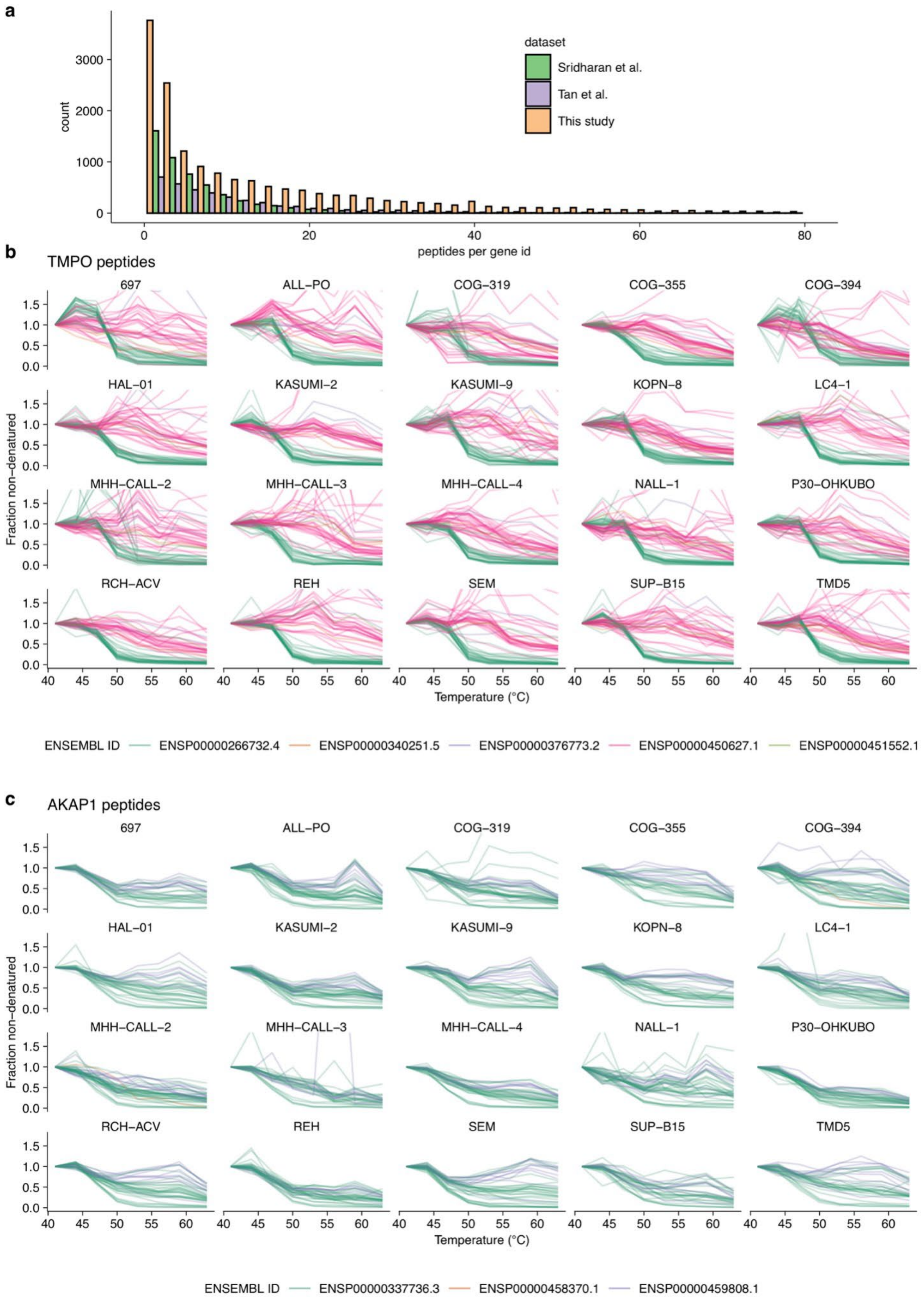

Supplementary Figure 2: **Gene level peptide analysis.** a) Comparison of unfiltered peptides mapping to gene symbols in this and previous studies. b, c) Melting profiles of peptides mapping to gene symbols resembling annotated proteoforms.

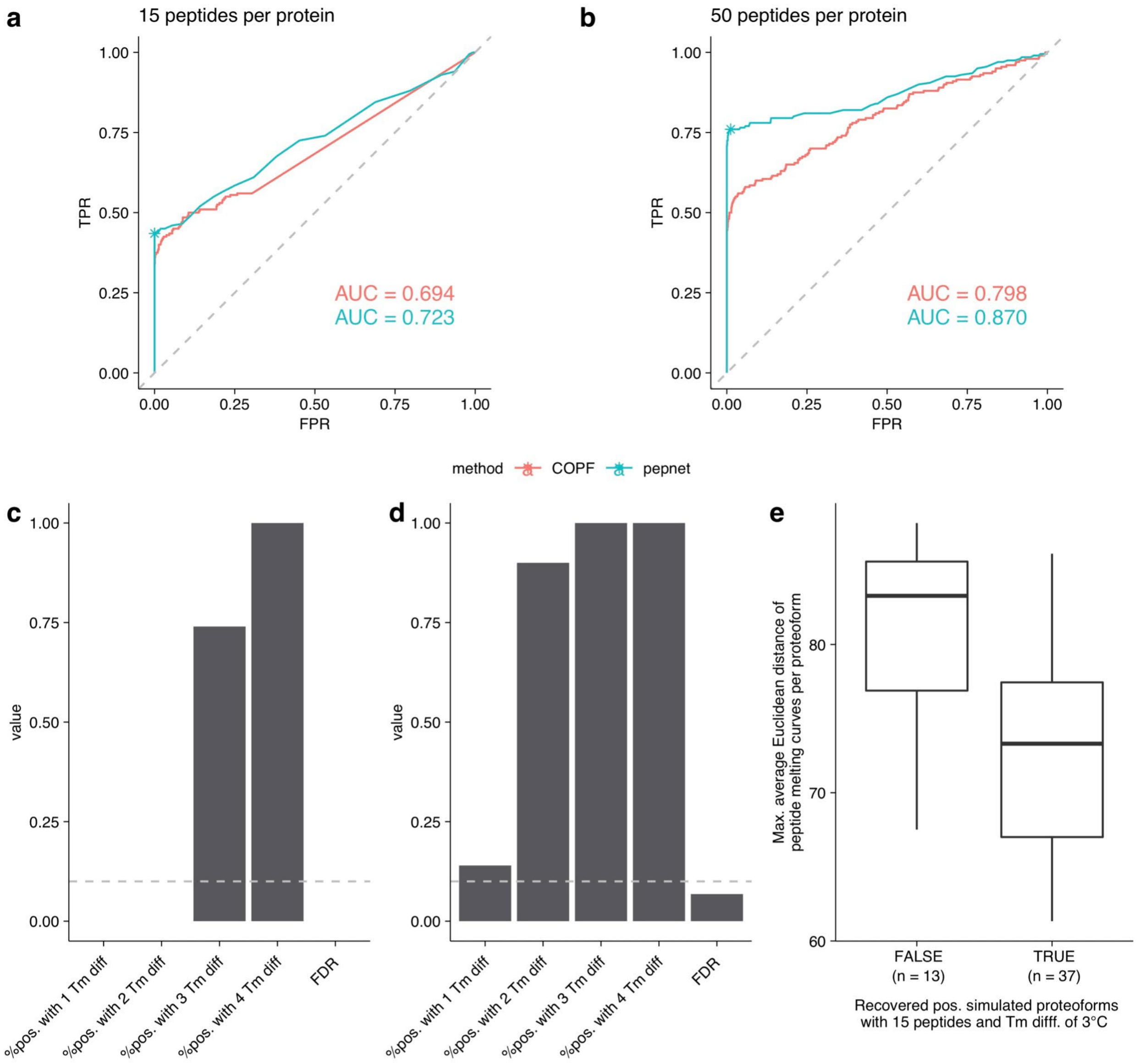

Supplementary Figure 3: Benchmark of the proteoform detection method on a simulated dataset. Receiver operating characteristic (ROC) curves obtained from applying the pepnet and COPF algorithms to a simulated dataset with 1000 true negatives and each 50 true positive proteoforms differing by 1, 2, 3 and 4°C in melting point. Performance plots for datasets simulated with a) 15 and b) 50 peptides per protein. Barplots showing the fraction of recovered positives with different Tm differences for the evaluation of the simulated datasets with c) 15 and d) 50 peptides. e) Boxplot comparing the maximal euclidean distance of peptides simulated to belong to one proteoform between positives that were detected and not detected by pepnet. Center lines represent the median, the bounds of the boxes are the 75 and 25% percentiles i.e., the interquartile range (IQR) and the whiskers correspond to the highest or lowest respective value or if the highest value is an outlier (greater than 1.5 * IQR from the bounds of the boxes) it is exactly 1.5 * IQR.

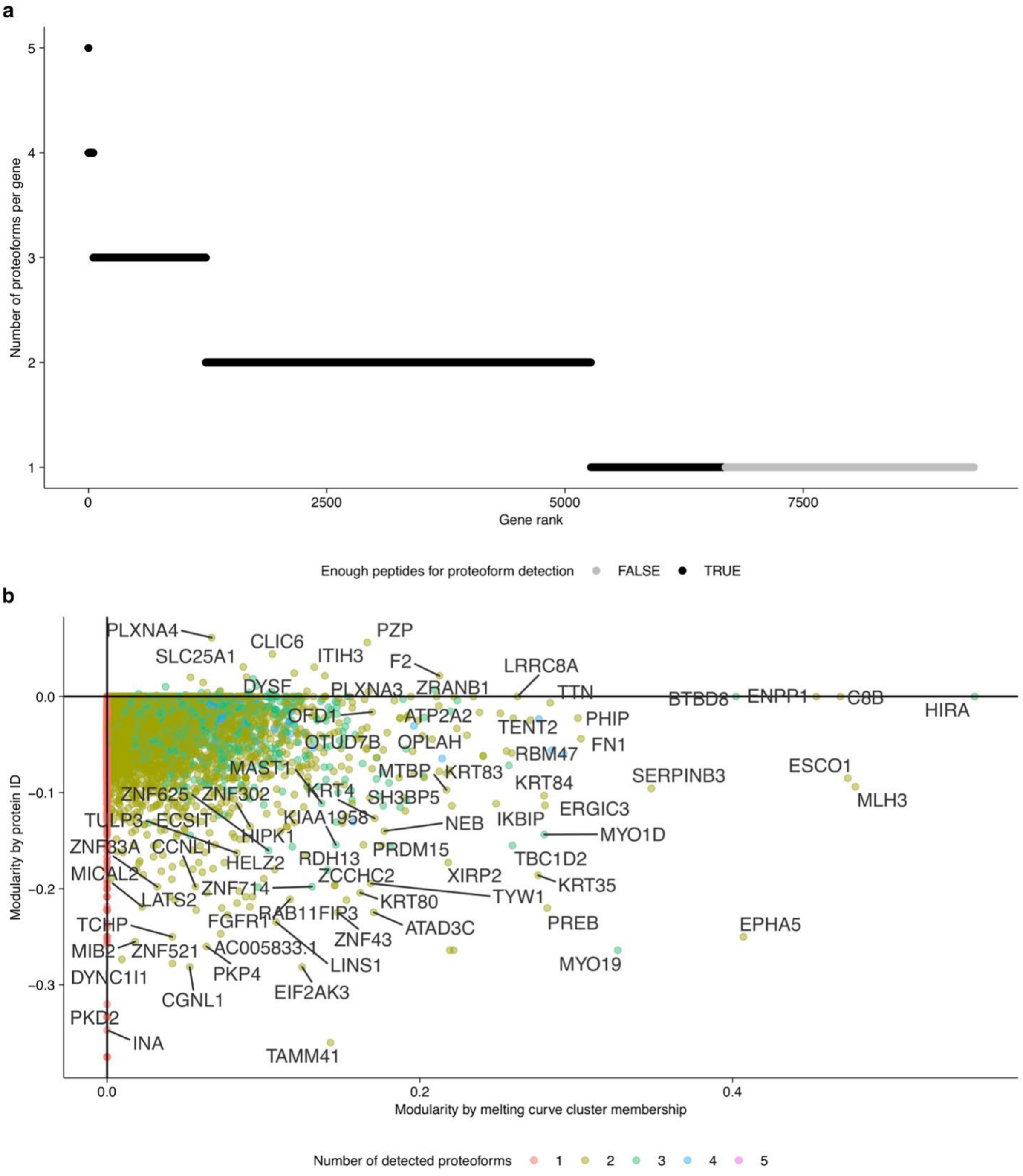

Supplementary Figure 4: **Proteoform detection statistics.** a) Dotplot of the number of detected proteoforms per gene symbol. b) Scatterplot of modularity by protein ID (ENSEMBL) versus modularity by detected proteoform based on melting curve similarity.

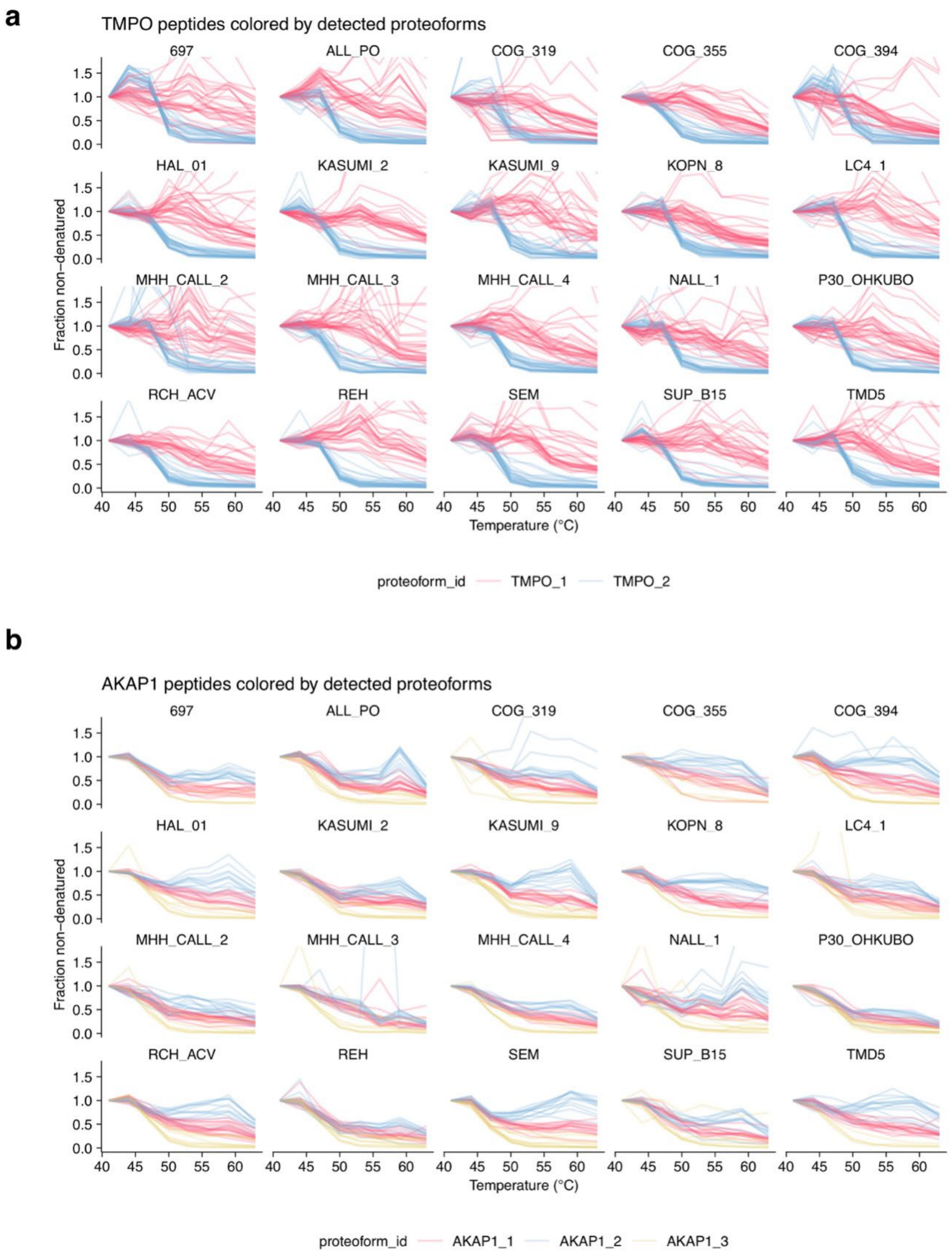

Supplementary Figure 5: Peptide profiles grouped by detected proteoforms. Melting profiles of peptides mapping to gene symbols colored by detected proteoform groups. a) TMPO and b) AKAP1.

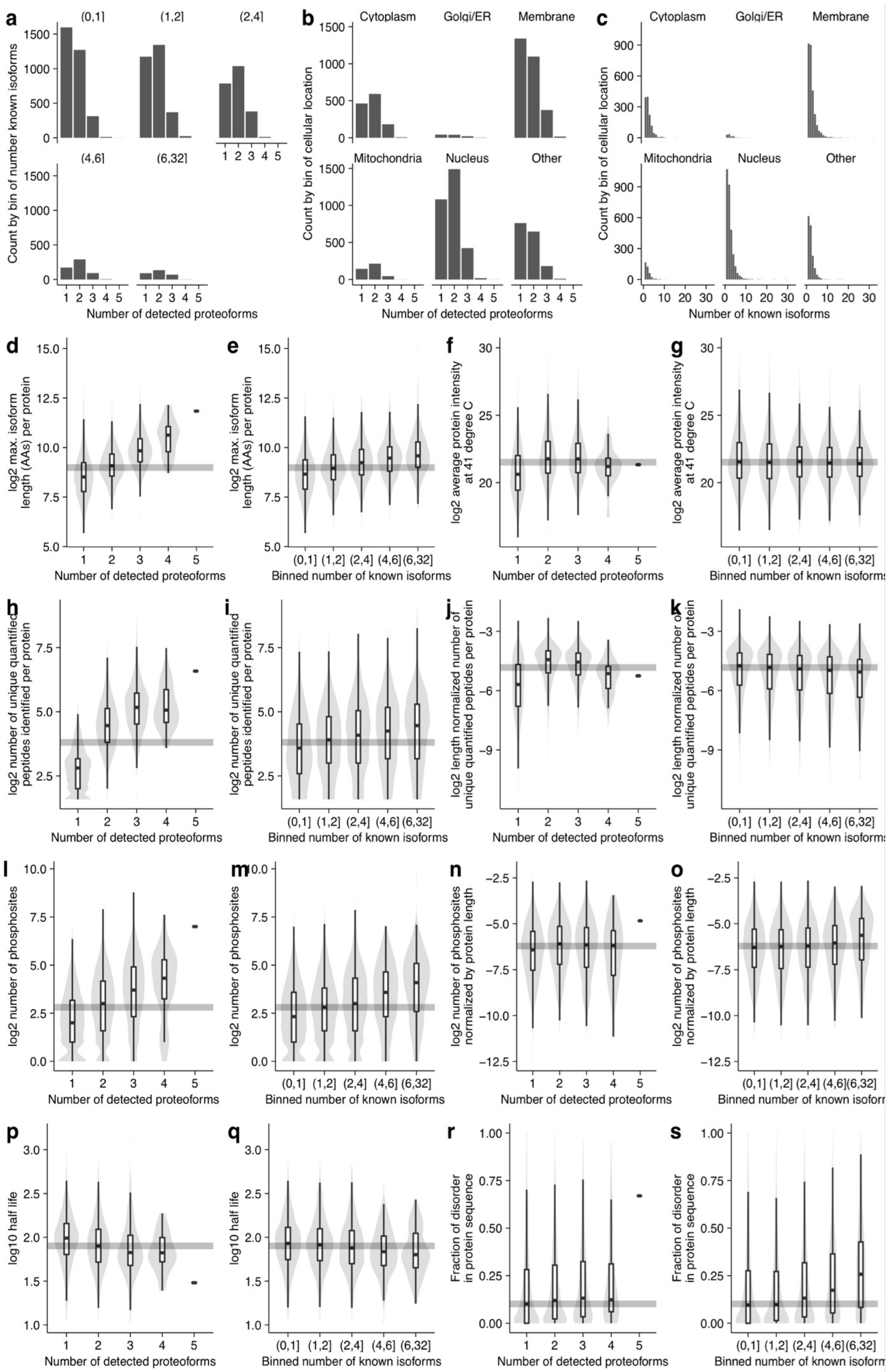

(Legend on next page)

Supplementary Figure 6: Features of proteins with detected proteoforms versus known isoforms. Barplots of the number of detected proteoforms per a) binned numbers of known isoforms per protein (n = 3193 (0,1], 2910 (1,2], 2216 (2,4], 558 (4,6] and 296 (6,32]) and b) cellular location (n = 1245 (Cytoplasm), 108 (Golgi/ER), 2829 (Membrane), 403 (Mitochondria), 3014 (Nucleus) and 1600 (Other)). c) Barplot of number of known isoforms per protein per cellular location (n = 1245 (Cytoplasm), 108 (Golgi/ER), 2829 (Membrane), 403 (Mitochondria), 3014 (Nucleus) and 1600 (Other)). Violin plots of length per d) number of detected proteforms (n = 3834 (1), 4080 (2), 1228 (3), 56 (4), 1 (5)) and e) number of known isoforms per protein (n = 3193 (0,1], 2910 (1,2], 2216 (2,4], 558 (4,6] and 296 (6,32]). Violin plots of average summed peptide per protein intensity for proteins with different f) number of detected proteoforms (n = 3834 (1), 4080 (2), 1228 (3), 56 (4), 1 (5)) and g) number of known isoforms (n = 3193 (0,1], 2910 (1,2], 2216 (2,4], 558 (4,6] and 296 (6,32]). Violin plots of the number of unique quantified peptides per protein for different h) number of detected proteoforms (n = 3834 (1), 4080 (2), 1228 (3), 56 (4), 1 (5)) and i) known number of isoforms per protein (n = 3193 (0,1], 2910 (1,2], 2216 (2,4], 558 (4,6] and 296 (6,32]). Violin plots of the number of unique quantified peptides per protein normalized by protein length for different j) number of detected proteoforms (n = 3834 (1), 4080 (2), 1228 (3), 56 (4), 1 (5)) and k) known number of isoforms (n = 3193 (0,1], 2910 (1,2], 2216 (2,4], 558 (4,6] and 296 (6,32]) . Violin plots of the number of reported phosphosites per protein for different l) number of detected proteoforms (n = 3834 (1), 4080 (2), 1228 (3), 56 (4), 1 (5)) and m) number of known isoforms per protein (n = 3193 (0,1], 2910 (1,2], 2216 (2,4], 558 (4,6] and 296 (6,32]) . Violin plots of the number of reported phosphosites per protein normalized by protein length for different n) number of detected proteoforms (n = 3834 (1), 4080 (2), 1228 (3), 56 (4), 1 (5)) and o) number of known isoforms per protein (n = 3193 (0,1], 2910 (1,2], 2216 (2,4], 558 (4,6] and 296 (6,32]). Violin plots of protein half-lives for proteins with different numbers of p) detected proteoforms (n = 3834 (1), 4080 (2), 1228 (3), 56 (4), 1 (5)) and q) known of isoforms (n = 3193 (0,1], 2910 (1,2], 2216 (2,4], 558 (4,6] and 296 (6,32]). Violin plots of fractions of protein disorder for proteins with different numbers of r) detected proteoforms (n = 3834 (1), 4080 (2), 1228 (3), 56 (4), 1 (5)) and s) known of isoforms (n = 3193 (0,1], 2910 (1,2], 2216 (2,4], 558 (4,6] and 296 (6,32]). Center lines in all box plots represent the median, the bounds of the boxes are the 75 and 25% percentiles i.e., the interquartile range (IQR) and the whiskers correspond to the highest or lowest respective value or if the highest value is an outlier (greater than 1.5 * IQR from the bounds of the boxes) it is exactly 1.5 * IQR. The transparent black line represents the global median.

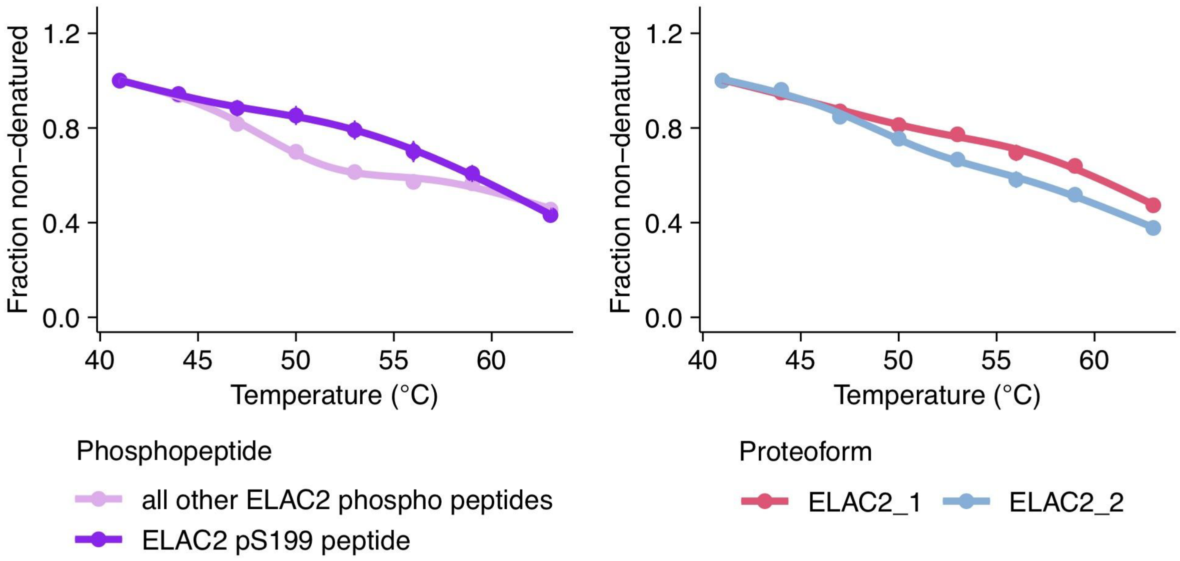

Supplementary Figure 7: **ELAC2 pS199 phosphopeptide melting.** a) Average melting profiles across cell lines (n = 20) for the ELAC2 peptide phosphorylated on serine 199 (detected in n = 18 cell lines) versus all other phosphopeptides found for ELAC2. b) Average melting profiles across cell lines (n = 20) for the two ELAC2 proteoforms. Error bars for all points represent the SEM.

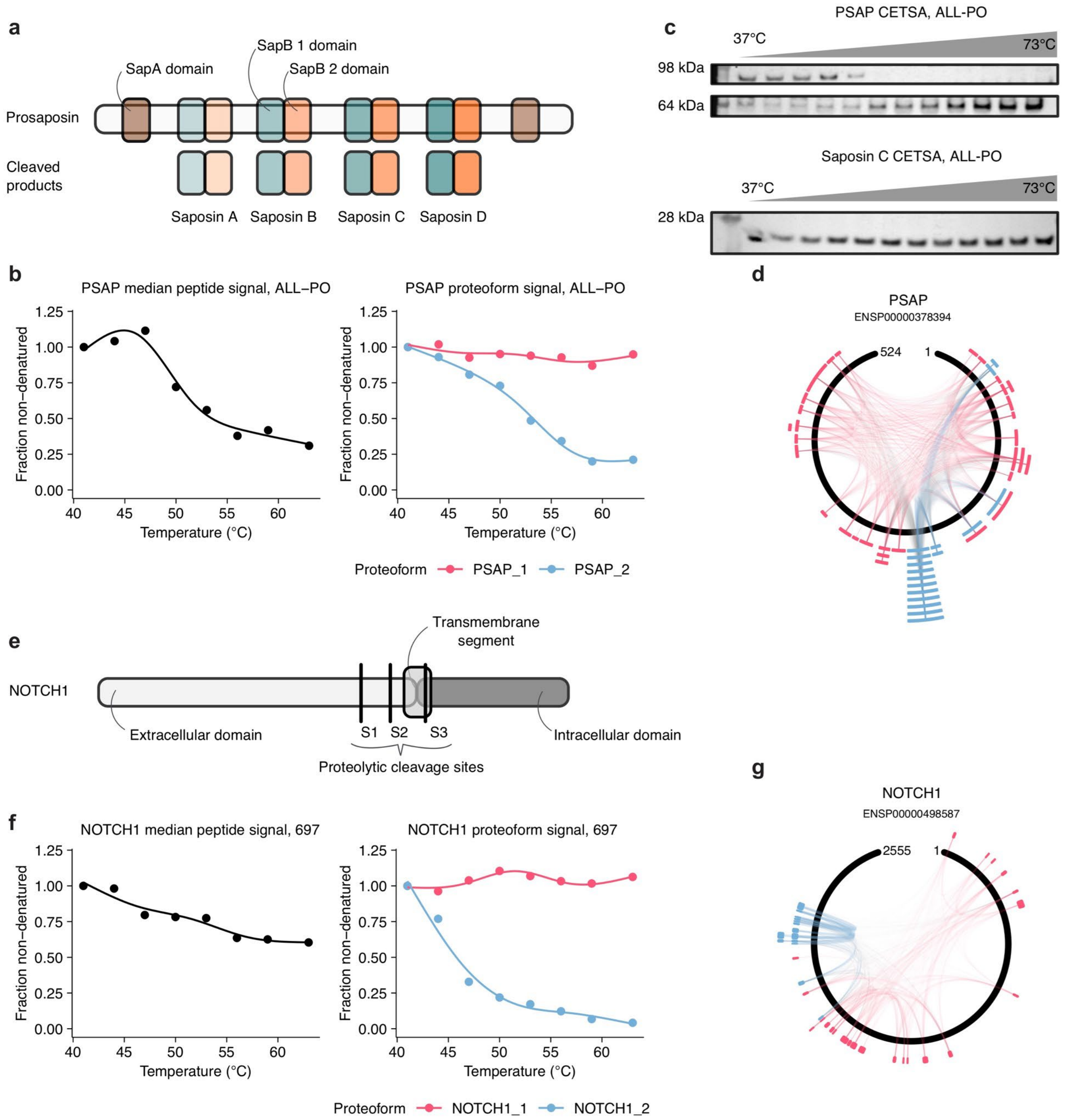

Supplementary Figure 8: **Proteoform examples indicative of proteolytic cleavage detected by similarity of peptide melting profiles.** a) Schematic of the protein domains of Pre-saposin (PSAP) and its cleaved products. PSAP is a protein which exists as an integral membrane protein, but also is proteolytically processed to form Saposin A, B, C and D [1]. b) Melting curves of the median peptide signal per PSAP gene symbol (left) and by detected proteoforms. c) A multi-band signal with differential thermal stabilities was also observed when performing a CETSA experiment (n = 1) with a PSAP antibody, whereas when using a Saposin C-specific antibody, solely a high thermal stability signal could be detected. When considering the mapping of the proteoform-specific peptides to the protein sequence (d), it became apparent that PSAP_1 likely reflected the non-cleaved form of PSAP. The peptides of PSAP_2, which featured lower thermal stability than those of PSAP_1, mapped to the second SapB domain and N-terminal of the

protein, suggesting that this proteoform could capture an intermediate cleavage product, since it is known that the processed Saposins exhibit high thermal stability [1] which is also reflected by the CETSA signal of Saposin C. e) Schematic of the protein domains of the transmembrane receptor NOTCH1. Upon ligand binding NOTCH1 releases a cleaved C-terminal domain which then functions as a transcriptional effector. f) Melting curves of the median peptide signal per NOTCH1 gene symbol (left) and by detected proteoforms. Considering the mapping of peptides assigned to the different proteoforms (g) revealed that NOTCH1_1 likely represented the membrane bound pool of the protein constituting high thermal stability, whereas NOTCH1_2 likely reflected the intracellular cleavage product.

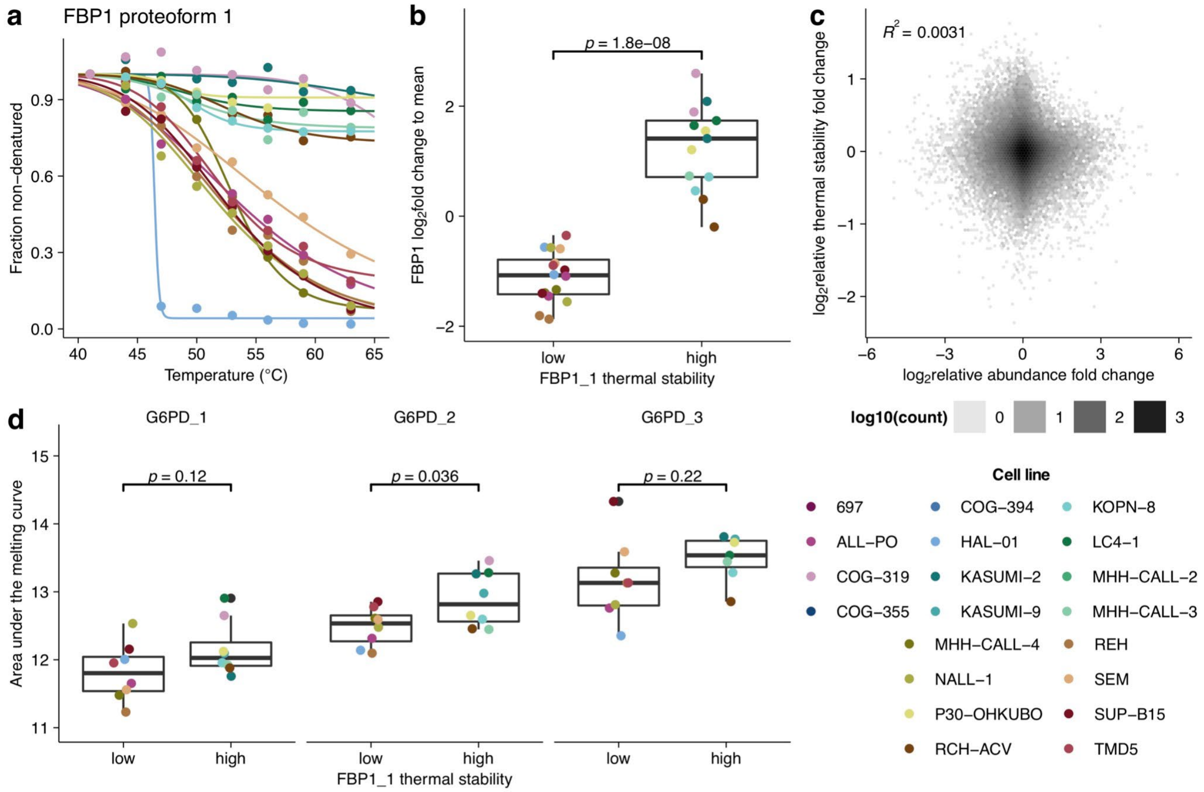

Supplementary Figure 9: **Thermal stability and abundance of FBP1.** a) Melting profiles of FBP1 proteoform 1 (FBP1_1) in the different cell lines. b) Boxplot of FBP1 $\log_2$ abundance fold change over mean in cell lines with low (n = 8) and high (n = 7) FBP1_1 thermal stability, technical replicate profiles are included. c) Global correlation of $\log_2$ thermal stability fold change over mean and $\log_2$ abundance fold change over mean across the cell lines. d) Boxplots of the $\log_2$ abundance fold changes over the mean of all G6PD proteoforms in cell lines with low (n = 8) and high (n = 8 for G6PD_1 and G6PD_2, n = 7 for G6PD_3) FBP1_1 thermal stability. Center lines in all box plots represent the median, the bounds of the boxes are the 75 and 25% percentiles i.e., the interquartile range (IQR) and the whiskers correspond to the highest or lowest respective value or if the highest value is an outlier (greater than 1.5 * IQR from the bounds of the boxes) it is exactly 1.5 * IQR. The p-values (p) were obtained from two-sided Welch two-sample t-tests.

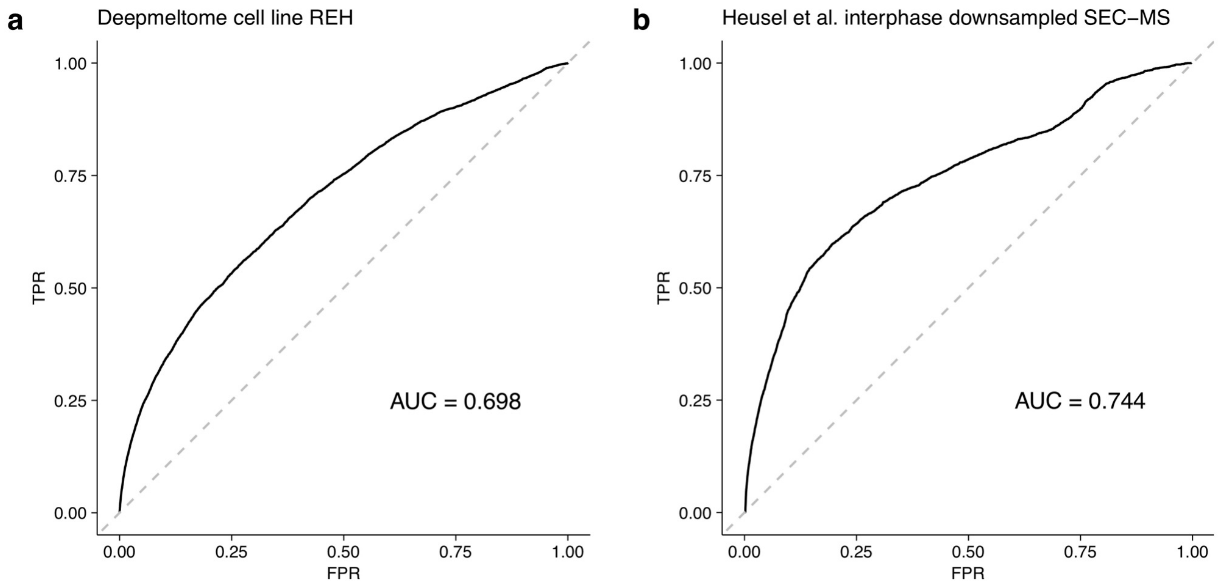

Supplementary Figure 10: Benchmark of PPI prediction of deep thermal proteome profiling versus size-exclusion chromatography coupled to MS (SEC-MS). Receiver operating characteristic (ROC) curves for the prediction of PPIs annotated by StringDB [2] (combined score >= 900) or direct and indirect interaction partners in protein complexes annotated by Ori et al. (2016) [3]. ROC curves obtained for a) Euclidean distances of fold changes of protein thermal stability (n = 8 per protein) relative to the lowest temperature (41°C) (shown is a random subset of 10,000 out of a total of 22,314,540 potential interactions) and b) Euclidean distances of fold changes of SEC-MS profiles (downsampled to n = 8 per protein) relative to the highest measurement across the profile (shown is a random subset of 10,000 out of a total of 9,805,806 potential interactions).

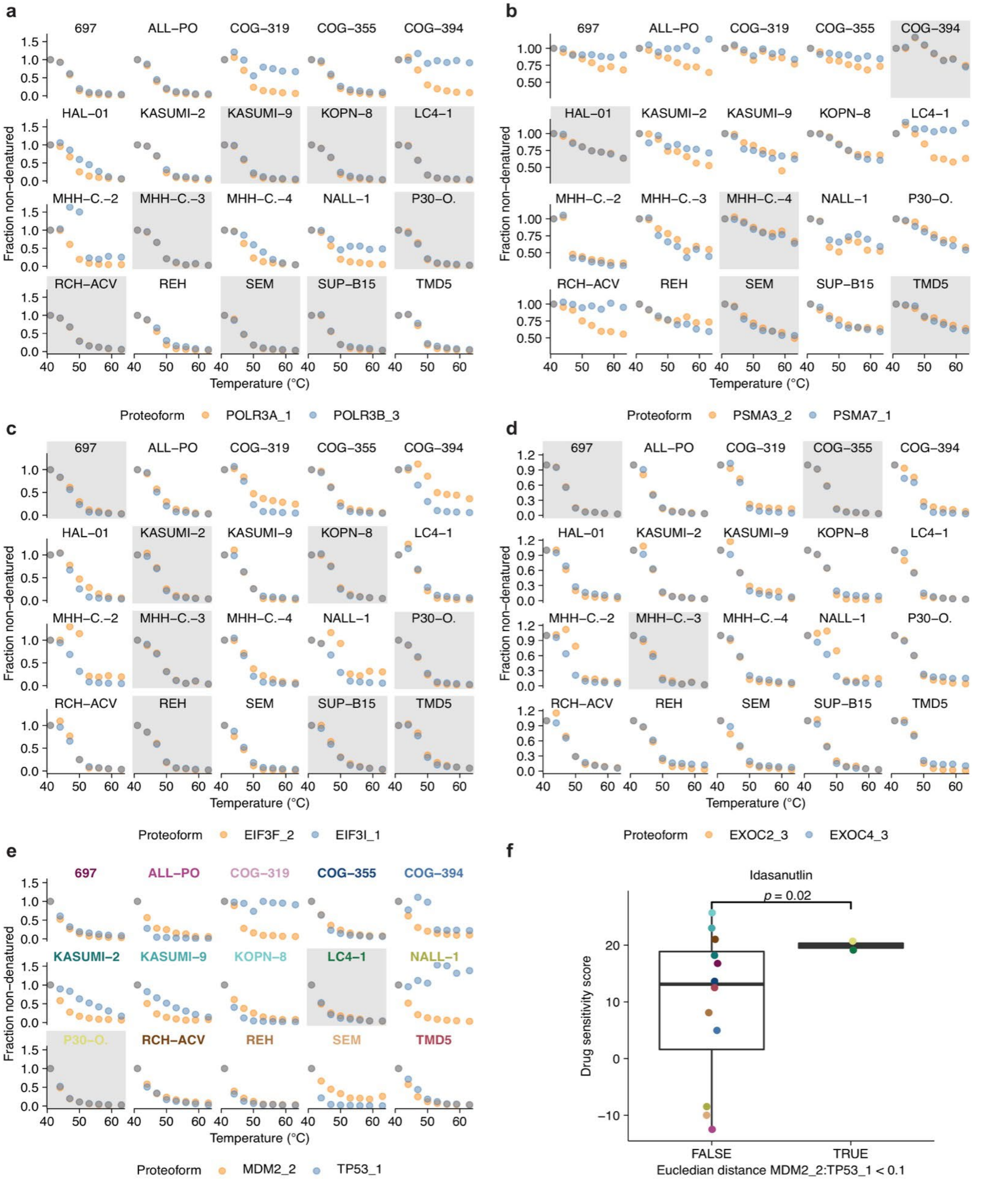

Supplementary Figure 11: **Additional profiles of pairs of proteoforms found to coaggregate differentially across cell lines.** Melting profiles of the differentially coaggregating proteoform pairs a) POLR3A_1 and POLR3B_3, b) PSMA3_2 and PSMA7_1, c) EIF3F_2 and EIF3I_1, d) EXOC2_3 and EXOC4_3 and e) MDM2_2 and TP53_1. Profiles indicative of coaggregation (Euclidean distance between profiles < 0.1) are shown with a gray background and otherwise with a white background. f) Boxplot of selective drug sensitivity scores (sDSS) for idasanutlin for cell

lines which do (n = 2) or do not (n = 17) show coaggregation of MDM2_2 and TP53_1. The p-value (*p*) was obtained from a two-sided Welch two-sample t-test. Center lines in all box plots represent the median, the bounds of the boxes are the 75 and 25% percentiles i.e., the interquartile range (IQR) and the whiskers correspond to the highest or lowest respective value.

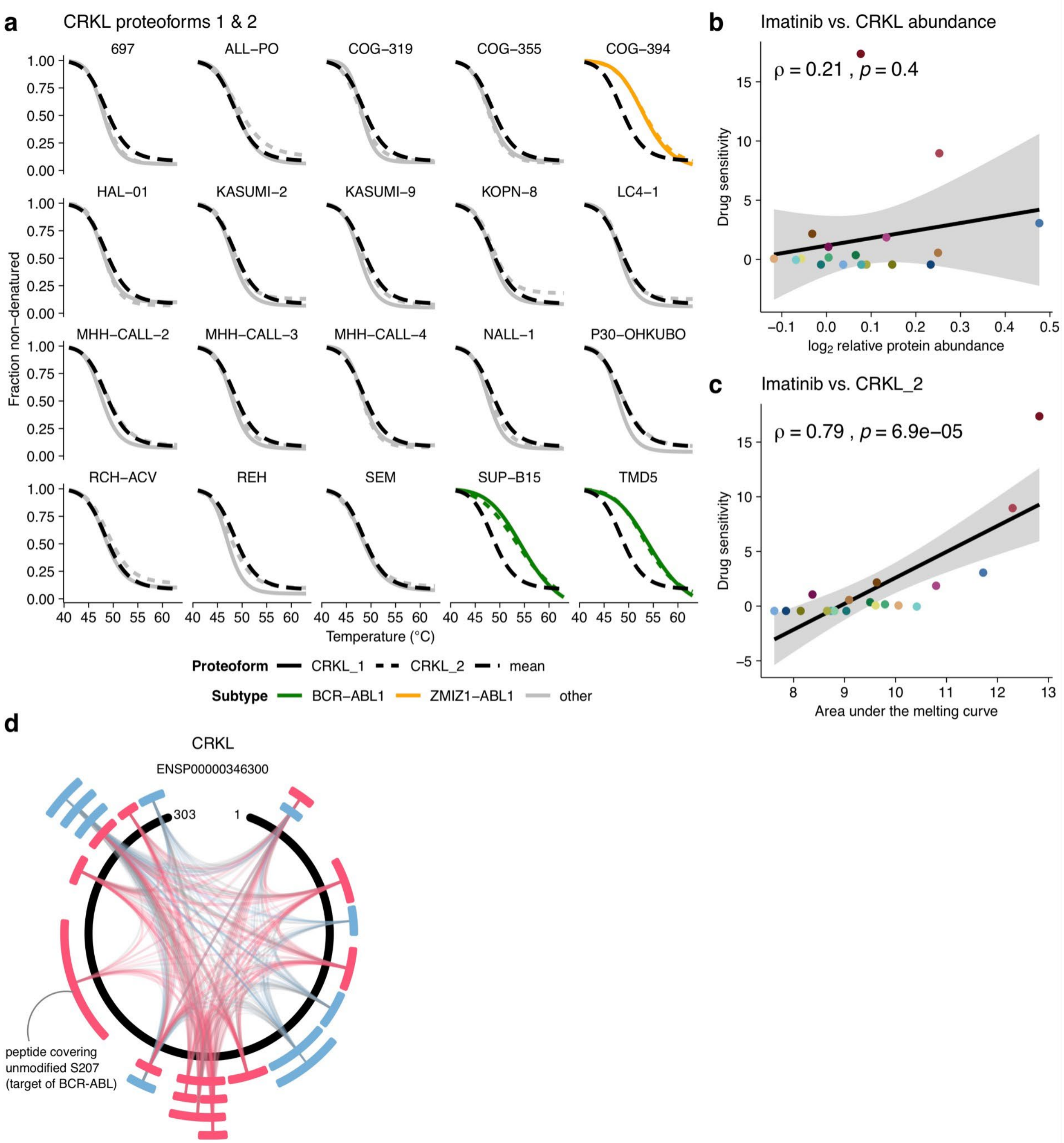

Supplementary Figure 12: **Correlation of CRKL thermal stability and abundance with drug sensitivity.** a) Melting profiles of CRKL proteoform 1 and 2 (CRKL_1 and CRKL_2) across cell lines. b) Correlation of CRKL abundance and sensitivity to imatinib. c) Correlation of CRKL_2 thermal stability and sensitivity to imatinib (sDSS). The Pearson correlation coefficient (ρ) and two-sided t-distribution p-values (*p*) are shown in each scatterplot. The linear regression trendline (black) and its 95% confidence interval (shaded gray area) are shown in the scatterplots. d) Peptide mapping to the CRKL sequence colored by proteoform assignment (CRKL_1: red, CRKL_2: blue).

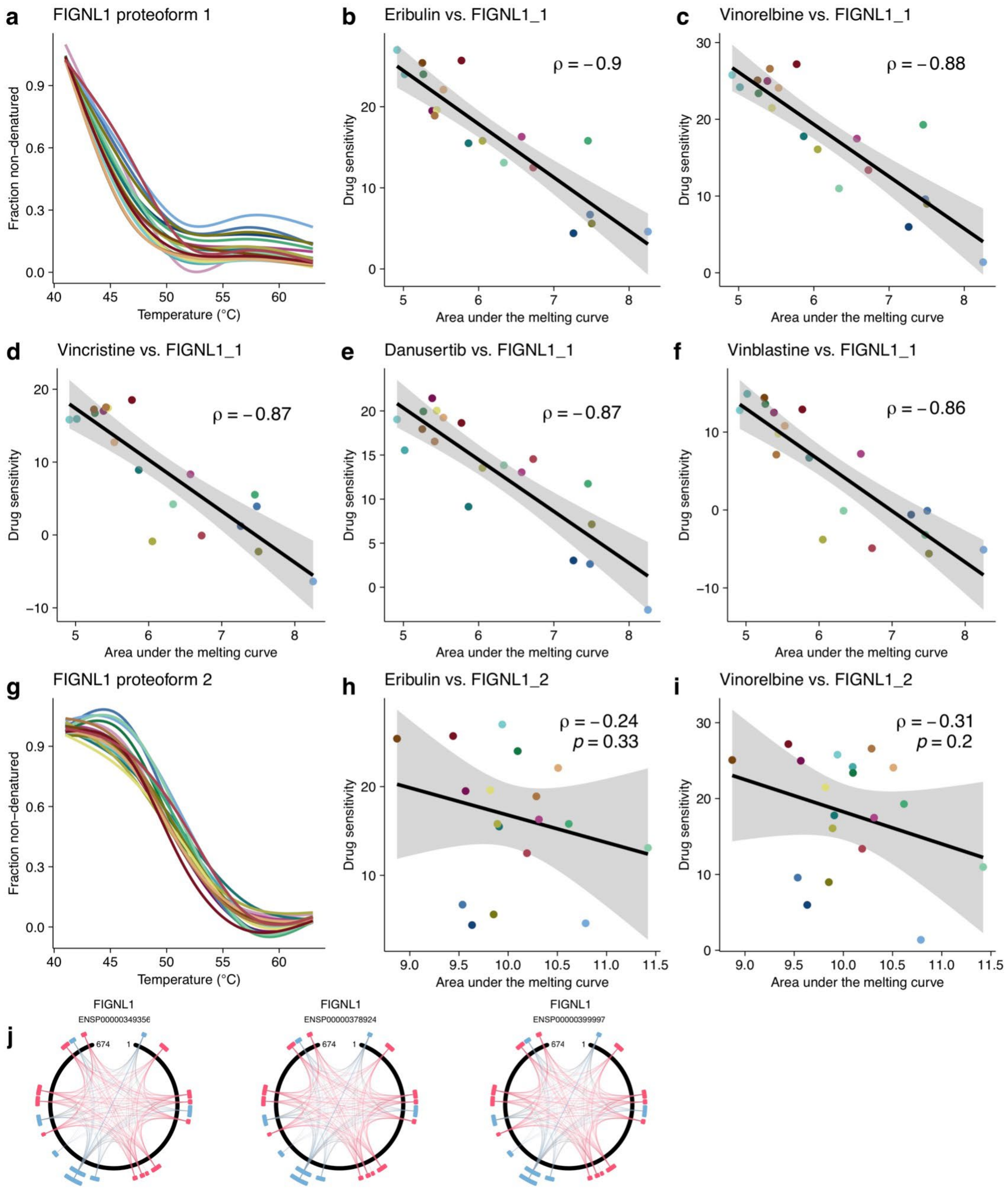

Supplementary Figure 13: **Correlation of FIGNL1 thermal stability with drug sensitivity (sDSS).** a) Melting profiles of FIGNL1 proteoform 1 (FIGNL1_1) across cell lines. Significant FIGNL1_1 thermal stability association with drug sensitivity to b) eribulin, c) vinorelbine, d) vincristine, e) danusertib and f) vinblastine. g) Melting profiles of FIGNL1 proteoform 2 (FIGNL1_2) across cell lines. Correlation of FIGNL1_2 thermal stability and sensitivity to h)

eribulin and i) vinorelbine. The Pearson correlation coefficient is represented by ρ and two-sided t-distribution p-values are represented by *p* in the scatterplots. The linear regression trendline (black) and its 95% confidence interval (shaded gray area) are also shown. j) Peptide mapping to different ENSEMBL protein ids of FIGNL1 colored by proteoform assignment (FIGNL1_1: red, FIGNL1_2: blue).

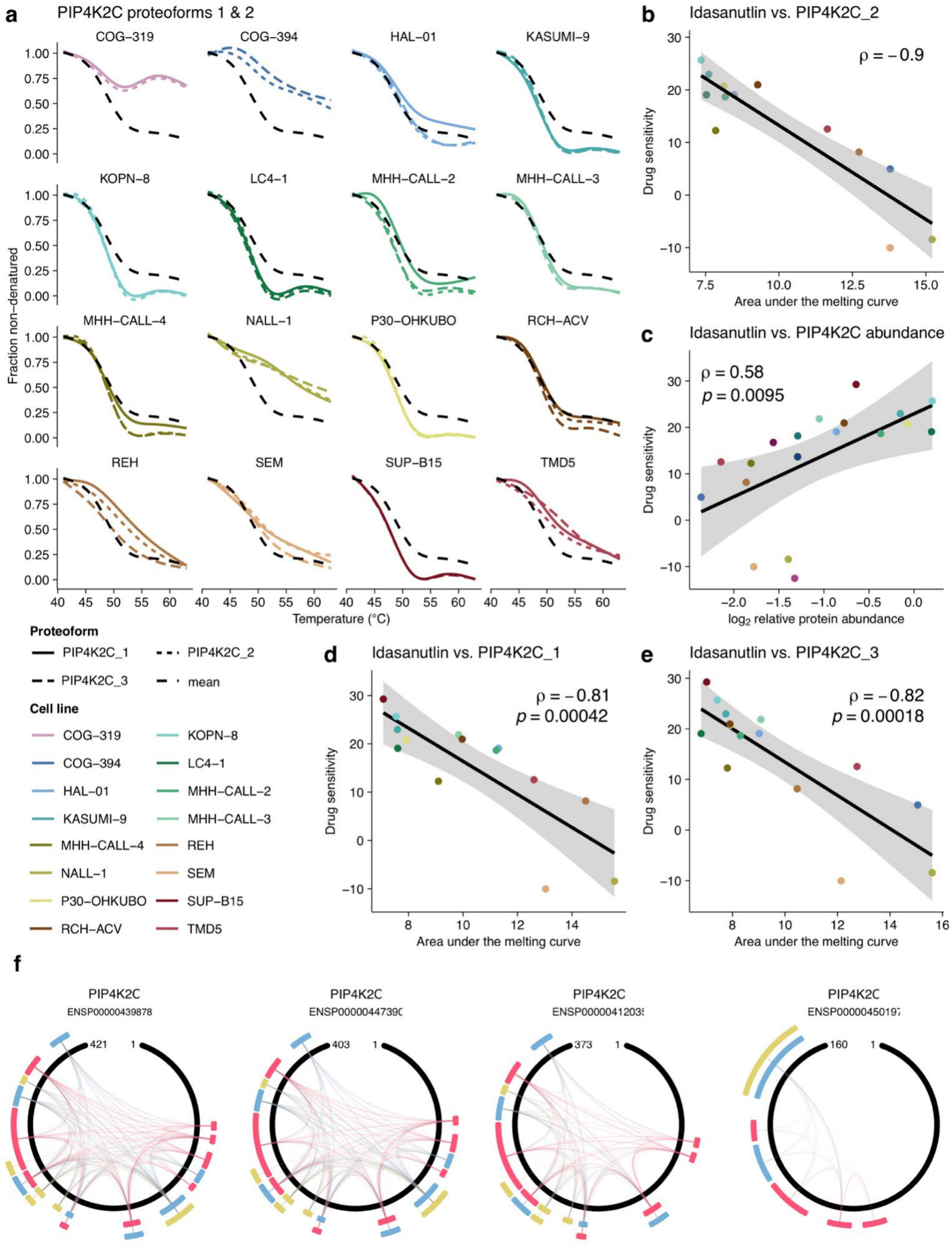

(Legend on next page)

Supplementary Figure 14: **Correlation of PIP4K2C thermal stability and abundance with drug sensitivity (sDSS).** a) Thermal stability profile of the PIP4K2C proteoforms. b) Scatterplot of sensitivity to idasanulin versus PIP4K2C_2 thermal stability. c) Correlation of idasanulin sensitivity versus PIP4K2C $\log_2$ abundance fold changes over the mean across cell lines. Scatterplots of sensitivity to idasanulin versus d) PIP4K2C_1 and e) PIP4K2C_3 thermal stability. The Pearson correlation coefficient (ρ) and two-sided t-distribution p-values (p) for the comparison are shown in each scatterplot. The linear regression trendline (black) and its 95% confidence interval (shaded gray area) are shown in the scatterplots. f) Peptide mapping to the four ENSEMBL protein ids with most mapped peptides of PIP4K2C colored by proteoform assignment (PIP4K2C_1: red, PIP4K2C_2: blue, PIP4K2C_3: yellow).

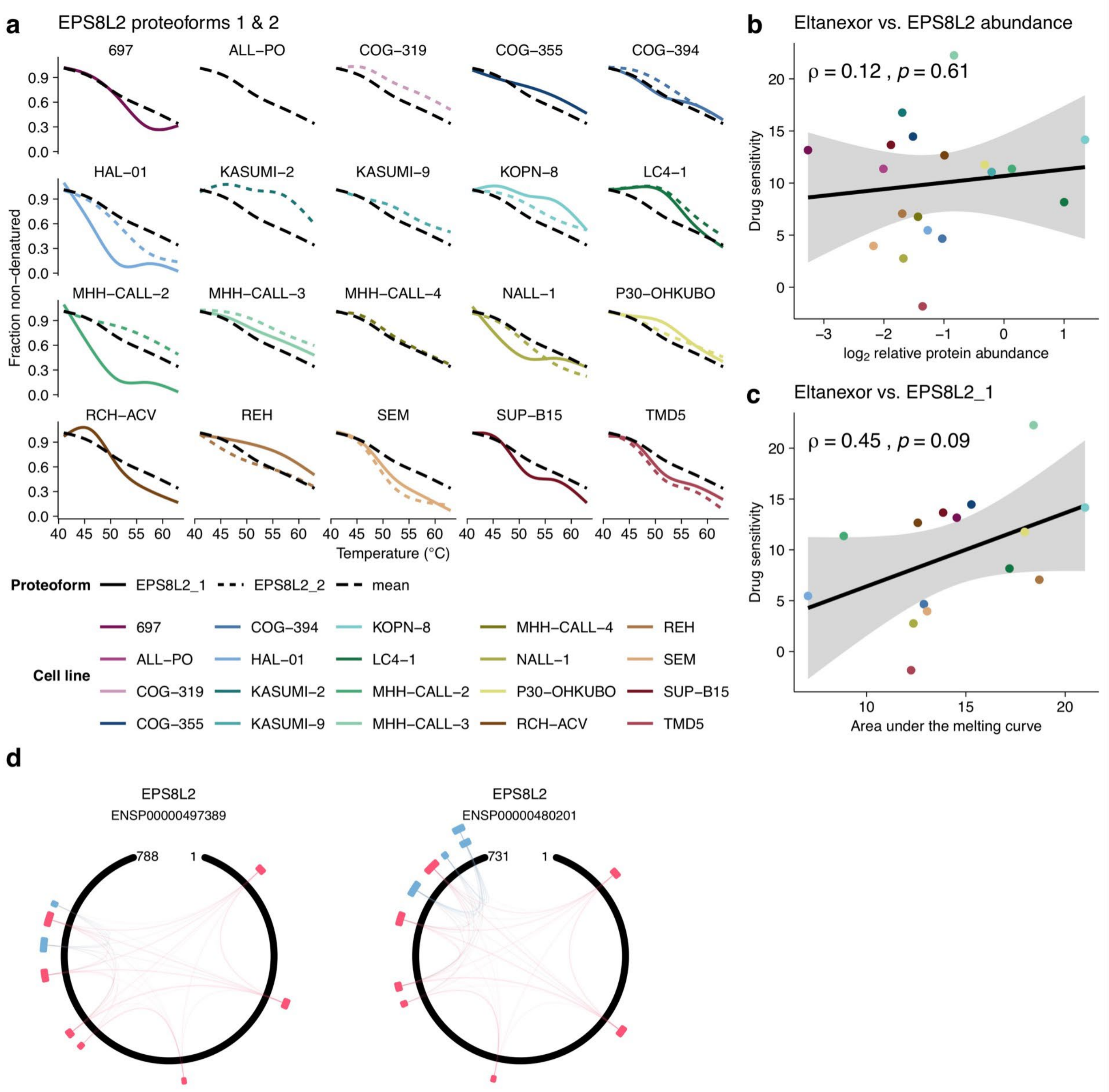

Supplementary Figure 15: **Correlation of EPS8L2 thermal stability and abundance with drug sensitivity (sDSS).** a) Thermal stability profile of the EPS8L2 proteoforms. b) Scatterplot of sensitivity to eltanexor versus EPS8L2 $\log_2$ abundance fold changes over the mean across cell lines. c) Scatterplot of sensitivity to eltanexor versus EPS8L2_1 thermal stability across cell lines. The Pearson correlation coefficient (ρ) and two-sided t-distribution p-values (*p*) for the comparison are shown in each scatterplot. The linear regression trendline (black) and its 95% confidence interval (shaded gray area) are shown in the scatterplots. d) Peptide mapping to different ENSEMBL protein ids of EPS8L2 colored by proteoform assignment (EPS8L2_1: red, EPS8L2_2: blue).

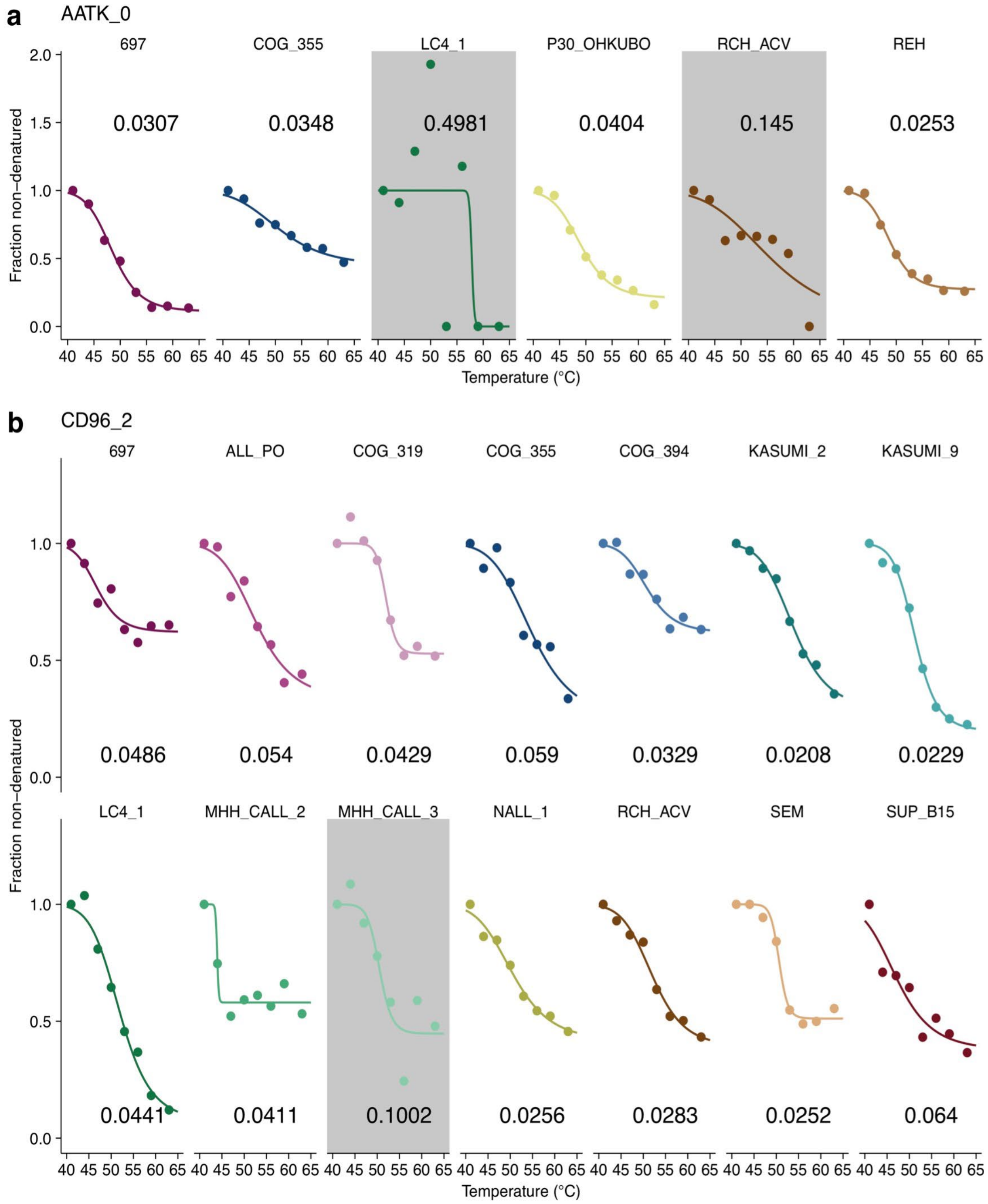

Supplementary Figure 16: **Illustration of the effect of filtering on the residual standard deviation of alternative model fits before computation of the F-statistic.** Melting profiles of a) AATK_0 and b) CD96_2 are shown. Numbers in subplots reflect residual standard deviation of alternative model fits in respective cell lines. Gray background color indicated that data for these cell lines has been filtered out from the analysis. In the case of AATK_0 the F-statistic with and without filtering changes from 1.28 to 25.22 respectively. In the case of CD96_2 the F-statistic with and without filtering changes from 7.86 to 10.33 respectively.

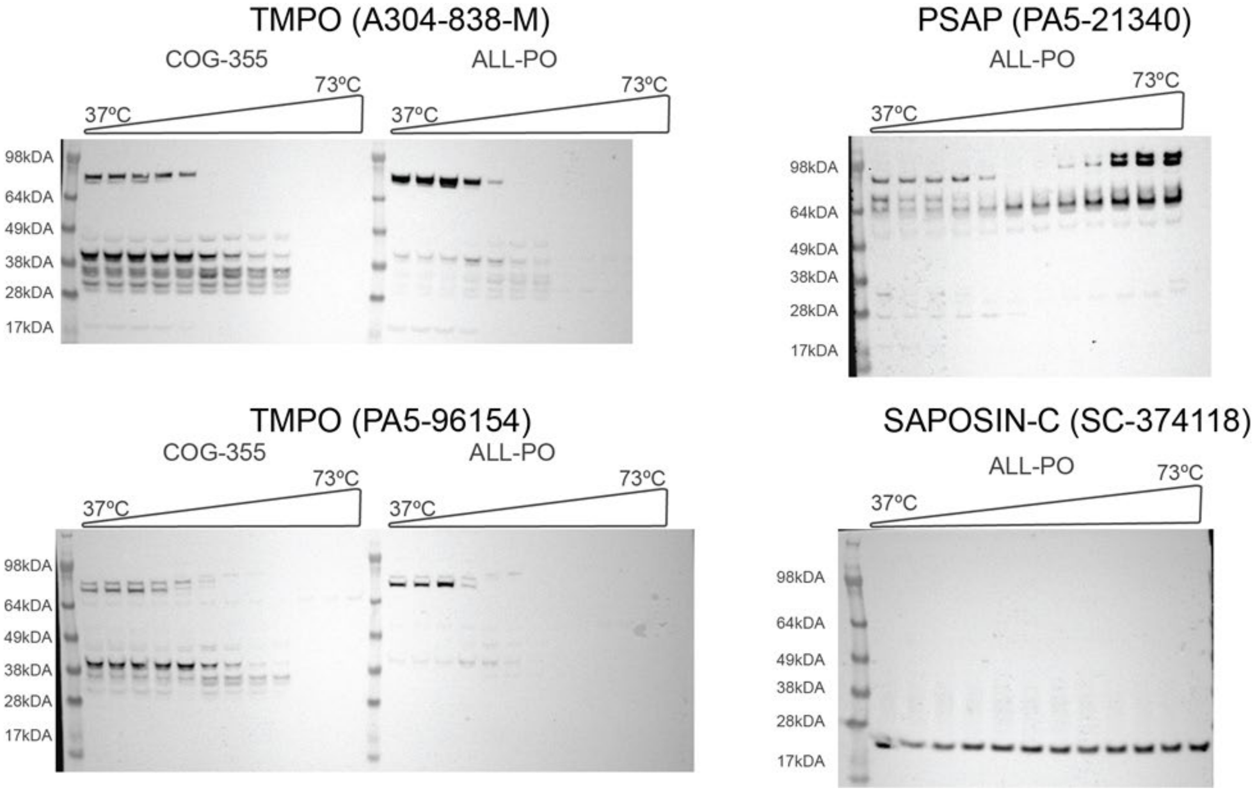

Supplementary Figure 17: Annotated uncropped western blot images from the CETSA experiments for TMPO in COG-355 and ALL-PO cell lines and PSAP in ALL-PO cell line. The CETSA temperature range (37-73°C) was performed once (n = 1) for each cell line. Bands for molecular weight markers (M.W., SeeBlue™Plus2) are annotated in each panel. The catalog nr for each antibody used is shown in brackets for each protein.

Supplementary References

1. Kishimoto, Y., Hiraiwa, M. & O'Brien, J. S. Saposins: structure, function, distribution, and molecular genetics. *J. Lipid Res.* **33**, 1255–1267 (1992).
2. Szklarczyk, D. *et al.* STRING v11: protein-protein association networks with increased coverage, supporting functional discovery in genome-wide experimental datasets. *Nucleic Acids Res.* **47**, D607–D613 (2019).
3. Ori, A. *et al.* Spatiotemporal variation of mammalian protein complex stoichiometries. *Genome Biol.* **17**, 47 (2016).
