# Supplementary material for: Deep thermal profiling for detection of functional proteoform groups
Source: Nat Chem Biol. 2023 Mar 20;19(8):962–71. doi: 10.1038/s41589-023-01284-8 (PMC10374440; doi:10.1038/s41589-023-01284-8)
Supplement: Supplementary file 2 — Reporting Summary [file 41589_2023_1284_MOESM2_ESM.pdf]

nature research

Corresponding author(s): Rozbeh Jafari, Mikhail Savitski

Last updated by author(s): Jan 23, 2023

Reporting Summary

Nature Research wishes to improve the reproducibility of the work that we publish. This form provides structure for consistency and transparency in reporting. For further information on Nature Research policies, see our Editorial Policies and the Editorial Policy Checklist.

Statistics

For all statistical analyses, confirm that the following items are present in the figure legend, table legend, main text, or Methods section.

n/a	Confirmed	
☐	☒	The exact sample size (*n*) for each experimental group/condition, given as a discrete number and unit of measurement
☐	☒	A statement on whether measurements were taken from distinct samples or whether the same sample was measured repeatedly
☐	☒	The statistical test(s) used AND whether they are one- or two-sided<br>*Only common tests should be described solely by name; describe more complex techniques in the Methods section.*
☐	☒	A description of all covariates tested
☐	☒	A description of any assumptions or corrections, such as tests of normality and adjustment for multiple comparisons
☐	☒	A full description of the statistical parameters including central tendency (e.g. means) or other basic estimates (e.g. regression coefficient) AND variation (e.g. standard deviation) or associated estimates of uncertainty (e.g. confidence intervals)
☐	☒	For null hypothesis testing, the test statistic (e.g. *F*, *t*, *r*) with confidence intervals, effect sizes, degrees of freedom and *P* value noted<br>*Give P values as exact values whenever suitable.*
☒	☐	For Bayesian analysis, information on the choice of priors and Markov chain Monte Carlo settings
☒	☐	For hierarchical and complex designs, identification of the appropriate level for tests and full reporting of outcomes
☐	☒	Estimates of effect sizes (e.g. Cohen's *d*, Pearson's *r*), indicating how they were calculated

Our web collection on statistics for biologists contains articles on many of the points above.

Software and code

Policy information about availability of computer code

Data collection	ProteoWizard tool suite (http://proteowizard.sourceforge.net/); MSGF+ v10072 (https://omics.pnl.gov/software/ms-gf); Percolator (v2.08); OpenMS project’s IsobaricAnalyzer (v2.0) (https://www.openms.de/openms220/); iBright Analysis Software (v5.0).
Data analysis	All data analysis code is available at: https://github.com/nkurzaw/deepPedAllMeltome, all code was run using R v. 4.0.0.

For manuscripts utilizing custom algorithms or software that are central to the research but not yet described in published literature, software must be made available to editors and reviewers. We strongly encourage code deposition in a community repository (e.g. GitHub). See the Nature Research guidelines for submitting code & software for further information.

Data

Policy information about availability of data

All manuscripts must include a data availability statement. This statement should provide the following information, where applicable:

- Accession codes, unique identifiers, or web links for publicly available datasets
- A list of figures that have associated raw data
- A description of any restrictions on data availability

All proteomics datasets have been deposited on PRIDE with the dataset identifier PXD031162. The post search files are also uploaded to Mendeley Data under the DOI: 10.17632/dwhtwh4dj7.1. Protein abundance data was taken from the PRIDE repository with the dataset identifier PXD023662. Protein-protein interactions were obtained from the public data download portal for STRING Database v11.5 (https://string-db.org/).

Field-specific reporting

Please select the one below that is the best fit for your research. If you are not sure, read the appropriate sections before making your selection.

☒ Life sciences ☐ Behavioural & social sciences ☐ Ecological, evolutionary & environmental sciences

For a reference copy of the document with all sections, see nature.com/documents/nr-reporting-summary-flat.pdf

Life sciences study design

All studies must disclose on these points even when the disclosure is negative.

Sample size	The number of cell lines included in the study (n=20) was chosen to include the largest attainable range of accessible cell lines meeting the following criteria:<br>- Acute lymphoblastic leukemia<br>- Childhood (age limit up to 20 years)<br>- BCP-ALL lineage, derived from sampling from any tissue (bone marrow, peripheral blood) and not limited to any subtypes (genes fusions, mutations).<br>- Commercial availability or easily available upon request from repositories<br>- 20 cell lines are sufficient to achieve large effect sizes, many significant comparisons, and robust biological replication for statistical analyses and method development.<br>-Based on previous publication (Jarzab et al. 2020, DOI: 10.1038/s41592-020-0801-4) sample size of 20 was sufficient to identify differential thermal stability across cell lines.
Data exclusions	Non-protein coding transcripts were excluded from the analyses of RNAseq data.
Replication	All proteoform results were supported by strict replicate detection standards. Successfully identified proteoforms were only considered if they met these replication standards, therefore all data interpreted represents successful technical replication. Proteoform identification was derived from melt curves quantified in at least two cell lines and inclusion of at least three unique peptides. Two duplicate biological replicate proteomics datasets were prepared from the cell lines RCH-ACV and MHH-CALL3. The replication attempts were successful (R2 = 0.79). Western blot experiments for selected cases were performed and replicated proteoform detection and melting. In addition, 23% of identified proteoforms are validated by current annotations in public data.
Randomization	The technical replicates were chosen at random prior to data analysis but excluding combinations of cell lines with the same genetic fusion subtype. Randomization was not otherwise relevant to this study because it generated a complete and comprehensive dataset representing all possible parameters that could be detected using data dependent mass spectrometry proteomics methods. The cell line panel as developed represents as many possible known and rare subtypes of childhood BCP-ALL that could be obtained from a readily available source, selection of these cell lines was not altered based on additional randomization criteria.
Blinding	Investigator blinding was not relevant to this study. All conclusions were obtained by or supported by unbiased non-parametric analyses, which represent in-depth results of many parameters obtained in a technically identical and unsupervised manner.

Reporting for specific materials, systems and methods

We require information from authors about some types of materials, experimental systems and methods used in many studies. Here, indicate whether each material, system or method listed is relevant to your study. If you are not sure if a list item applies to your research, read the appropriate section before selecting a response.

Materials & experimental systems

n/a	Involved in the study	
☐	☒	Antibodies
☐	☒	Eukaryotic cell lines
☒	☐	Palaeontology and archaeology
☒	☐	Animals and other organisms
☒	☐	Human research participants
☒	☐	Clinical data
☒	☐	Dual use research of concern

Methods

n/a	Involved in the study	
☒	☐	ChIP-seq
☒	☐	Flow cytometry
☒	☐	MRI-based neuroimaging

Antibodies

Antibodies used	TMPO/LAP2 (Thermo Fisher Scientific, cat. No; A304-838A-M, RRID: AB_2782213 and cat# PA5-96154, RRID:AB_2807956 both at 1:1000 dilution), PSAP (Thermo Fisher Scientific, cat# PA5-21340, RRID: AB_11154619, 1:1000 dilution), Saposin-C (Santa Cruz Biotechnology cat# sc-374119, RRID: AB_10947406, 1:500 dilution), Anti-mouse secondary (Millipore, cat no. AP127P, RRID: AB_92472, 1:5000 dilution), Anti-rabbit secondary (Santa Cruz Biotechnology cat# sc-2004, RRID: AB_631746, 1:5000 dilution)

Validation

Protein size and thermal properties were in alignment with the results obtained in our mass spectrometry dataset. Advanced verification by knockdown was performed by the antibody vendor for: PSAP (Thermo Fisher Scientific, cat# PA5-21340, RRID: AB_11154619). No advanced verification was reported by vendor for the remainder of antibodies.

Eukaryotic cell lines

Policy information about cell lines

Cell line source(s)

ALL-PO BBCF/iclc.it; COG-319; COG-355; COG-394 COG; HAL-01 DSMZ; KASUMI-2 DSMZ; KASUMI-9 JCRB; KOPN-8 DSMZ; LC4-1 JCRB; MHH-CALL-2 DSMZ; MHH-CALL-3 DSMZ; MHH-CALL-4 DSMZ; NALL-1 JCRB; P30-OHKUBO DSMZ; RCH-ACV DSMZ; REH ATCC; SEM DSMZ; SUP-B15 ATCC; TMD5 JCRB; 697 DSMZ.

Authentication

All cell lines were authenticated by STR profiling (Eurofins Genomics, Ebersberg, Germany).

Mycoplasma contamination

All cell lines were tested for Mycoplasma by MycoAlert Mycoplasma detection kit (Lonza). All cell lines used in this study tested negative for mycoplasma.

Commonly misidentified lines
(See ICLAC register)

None
